# Interaction between Extreme Temperature Events and Fine Particulate Matter on Cardiometabolic Multimorbidity: Evidence from Four National Cohort Studies

**DOI:** 10.1021/acs.est.4c02080

**Published:** 2024-07-03

**Authors:** Shouxin Peng, Zhaoyuan Li, John S. Ji, Bingbing Chen, Xiaoyi Yin, Wei Zhang, Feifei Liu, Huanfeng Shen, Hao Xiang

**Affiliations:** † Global Health Department, School of Public Health, Wuhan University, Wuhan 430071, China; ‡ Global Health Institute, Wuhan University, Wuhan 430071, China; § Vanke School of Public Health, Tsinghua University, Beijing 100084, China; ∥ School of Resource and Environmental Sciences, Wuhan University, Wuhan 430079, China

**Keywords:** Extreme temperature events, Fine particulate matter with constituents, Cardiometabolic multimorbidity, Interaction effect, Multicohort study

## Abstract

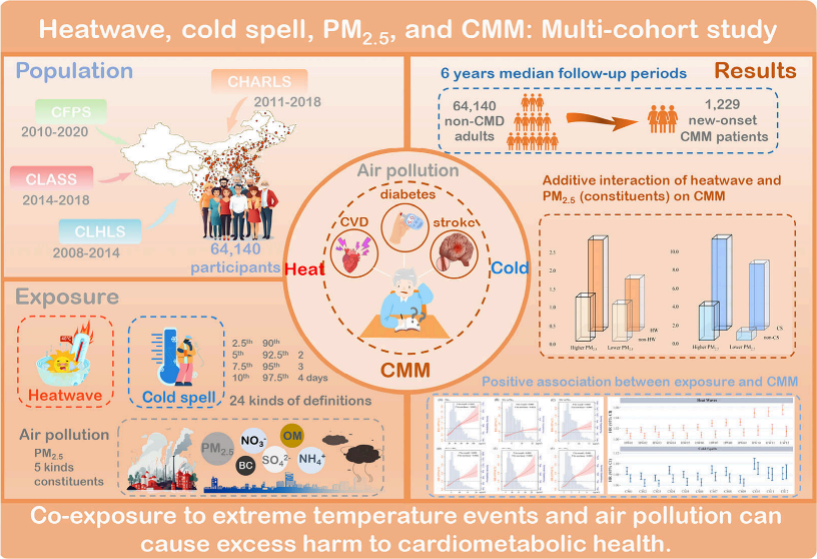

Accumulating evidence linked extreme temperature events (ETEs) and fine particulate matter (PM_2.5_) to cardiometabolic multimorbidity (CMM); however, it remained unknown if and how ETEs and PM_2.5_ interact to trigger CMM occurrence. Merging four Chinese national cohorts with 64,140 free-CMM adults, we provided strong evidence among ETEs, PM_2.5_ exposure, and CMM occurrence. Performing Cox hazards regression models along with additive interaction analyses, we found that the hazards ratio (HRs) of CMM occurrence associated with heatwave and cold spell were 1.006–1.019 and 1.063–1.091, respectively. Each 10 μg/m^3^ increment of PM_2.5_ concentration was associated with 17.9% (95% confidence interval: 13.9–22.0%) increased risk of CMM. Similar adverse effects were also found among PM_2.5_ constituents of nitrate, organic matter, sulfate, ammonium, and black carbon. We observed a synergetic interaction of heatwave and PM_2.5_ pollution on CMM occurrence with relative excess risk due to the interaction of 0.999 (0.663–1.334). Our study provides novel evidence that both ETEs and PM_2.5_ exposure were positively associated with CMM occurrence, and the heatwave interacts synergistically with PM_2.5_ to trigger CMM.

## Abstract 2

We revealed that coexposure to extreme temperature and air pollution can cause excess harm to cardiometabolic health.

## 1 Introduction

Cardiometabolic multimorbidity (CMM) is simultaneous suffering from multiple cardiometabolic diseases (CMDs),^1,2^ typically involving heart disease, diabetes, and stroke. According to the WHO, the leading causes of global mortality for CMD are estimated to be ischemic heart disease (1st), stroke (2nd), and diabetes (9th).^3^ Importantly, patients with CMM face multiplicative risk of mortality compared with single CMDs.^4,5^ In China, the prevalence of CMM was reported as 11.6–16.9%,^6,7^ which poses a substantial burden on both families and societies. Even worse, China’s aging population and rising incidence of cardiometabolic disorders might lead to a large increase in CMM patients.^6,8^ Therefore, identifying modifiable risk factors of CMM can guide public health preparedness. Although several risk factors, such as obesity, smoking, and sedentary behaviors, have been well-established,^9,10^ specific causes of more CMM patients remain unclear.

Emerging studies suggested a positive association between air pollution, especially fine particulate matter (PM_2.5_), and single CMDs.^11,12^ The GBD reports estimated that PM_2.5_ pollution contributed significantly to burdens of CMDs death, accounting for 14.07% of ischemic heart disease, 13.33% of diabetes, and 16.94% of stroke, respectively.^13^ However, existing evidence for the association between PM_2.5_ and CMM has yet to be fully accounted for. To the best of our knowledge, only a few studies have examined this topic.^7,14,15^ Specifically, the Urban and Rural Elderly Population (UREP) survey observed that per 10 μg/m^3^ increment in PM_2.5_ concentrations was associated with 2.2%–7.6% higher risk of CMM.^7^ Similarly, findings from the UK Biobank also reported a positive association between PM_2.5_ exposure and CMDs, as well as the progression to CMM.^14^ Additionally, PM_2.5_ is a complex mixture comprising various chemical constituents, mainly including nitrate (NO_3_^–^), organic matter (OM), sulfate (SO_4_^2–^), ammonium (NH_4_^+^), and black carbon (BC), etc. These constituents have varying health effects and can indicate the emission sources.^16,17^ It is therefore crucial to identify the key toxic constituents of total PM_2.5_ mass to develop targeted environmental control strategies. However, there is currently a lack of evidence regarding the associations between PM_2.5_ constituents and CMM.

Amidst climate change, extreme temperature events (ETEs) like heatwave and cold spell are occurring more frequently, presenting a substantial threat to human health. The Multi-Country Multi-City Collaborative Network study revealed that exposure to heat and cold was linked to an elevated cardiovascular mortality, accounting for 0.22% (95%CI: 0.21–0.23) and 0.91% (0.89–0.92) excess death, respectively.^18^ Given the previous evidence on single CMDs,^19−21^ we hypothesize that heatwave and cold spell might also impose adverse effect on CMM, despite a lack of direct evidence currently. Another critical issue was the joint effect of ETEs and PM_2.5_ pollution. Climate change could trigger events such as wildfire and sandstorms, exacerbating PM_2.5_ pollution levels.^22^ Additionally, ETEs, particularly heatwave, could accelerate respiratory rates and affect ventilation, resulting in increased inhalation of pollutants.^23^ Recent evidence indicated that both heatwave and cold spell interact synergistically with PM_2.5_ pollution, resulting in an increased risk of circulatory mortality.^24,25^ However, whether ETEs, PM_2.5_, and its constituents work together synergistically to heighten the risk of CMM remains unknown.

To address these knowledge gaps, we aimed to assess the independent and interaction effects of heatwave, cold spell, and PM_2.5_ exposure to the risk of CMM using current national cohorts in China. Our findings will contribute evidence regarding the impact of climate change and air pollution on human cardiometabolic health.

## 2 Materials and Methods

### 2.1 Study Population

Participants’ information was derived from four large-scale nationally representative surveys, involving China Health and Retirement Longitudinal Study (CHARLS), China Longitudinal Aging Social Survey (CLASS), China Family Panel Studies (CFPS), and Chinese Longitudinal Healthy Longevity Study (CLHLS). These national prospective cohorts covered 262 cities in 30 Chinese provinces. Details for the above evious studies (eMethods 1).^26−29^ Briefly, the four national surveys were all dynamic cohorts, conducting face-to-face personal interviews with baseline participants and newly enrolled individuals during follow-up visits. CHARLS recruited a total of 25,586 adults over 45 years, with a baseline survey (2011–2012) and three follow-up visits in 2013, 2015, and 2018. CLASS initially enrolled 11,511 participants aged over 60 years in 2014, followed by two subsequent visits in 2016 and 2018. CFPS, following a baseline survey in 2010, cumulatively included 57,945 participants aged above 18 years and followed every two years until 2020. CLHLS commenced in 1998 and conducted seven rounds of follow-up surveys with an interval of 2–3 years over the subsequent 20 years. Ultimately, we included 64,140 participants free of CMDs after excluding those with missing key information (Figure S1). Among these participants, 11,136 participants were from CHARLS (2011–2018), 7368 from CLASS (2014–2018), 38,476 from CFPS (2010–2020), and 7160 from CLHLS (2008–2014). Written informal consent was obtained from the participants during the interview. Institutional Review Boards of Peking University and Renmin University of China have approved the research protocol.

### 2.2 Exposure Assessment

Meteorological data was accessed from the National Center for Environmental Information (NCEI) (Figure S2). Specifically, we retrieved the daily average temperature (AT) from each NCEI station. We then employed the inverse distance weighted method to interpolate the national raster data set of daily AT. Accounting for the regional-climate variations, we defined ETEs by intensity and duration, based on a relative threshold approach.^30,31^ Currently, heat or cold cutoff values were determined as the 2.5th, 5th, 7.5th, 10th, 90th, 92.5th, 95th, and 97.5th percentiles of the daily AT for each city during study periods (2008–2020). ETEs were identified as consecutive days with daily AT higher than heat cutoff or lower than cold cutoff values for 2, 3, or 4 consecutive days (Table S1). We then calculated the ETEs day frequency,^32,33^ including heatwave and cold spell day frequency, as the total number of occurred-events days.

PM_2.5_ concentration along with 5 major chemical constituents, was derived from the Tracking Air Pollution in China data set,^34,35^ with a 10 km spatial resolution at a monthly level (2010–2020). Briefly, PM_2.5_ concentrations were estimated by a two-stage machine learning model that combined multiple-source information from ground observations, satellite AOD, operational CMAQ, and other ancillary data.^34^ Concentrations of constituents (NO_3_^–^, OM, SO_4_^2–^, NH_4_^+^, and BC) were obtained from operational CMAQ simulations using the above PM_2.5_ concentrations as the overall constraint.^35^ This data set had high accuracy with a cross-validation coefficient of determination of 0.64–0.83 in China. All pollutant concentrations were calculated as the city-level based on participants’ residence address with a one-year average before the occurrence of the outcome or the end of the study (eMethods 2 and 4).

### 2.3 CMM Definition

Participants were identified as CMM if they experienced at least two concurrent conditions among heart disease, stroke, and diabetes.^36^ The above physician-diagnosed disease was ascertained by self-reported health condition in the following questions. Heart disease status was determined by the question: “Have you ever been diagnosed with heart attack, coronary heart disease, angina, congestive heart failure, or other heart problems by a doctor?”. Stroke status was determined by the question: “Have you ever been diagnosed with stroke by a doctor?”. Diabetes status was determined by the question: “Have you ever been diagnosed with diabetes or high blood sugar?”. A detailed explanation was supplied in the eMethods 3.

### 2.4 Covariates

Covariates were thoughtfully chosen based on existing evidence.^7,14,36^ Prior-selected continuous covariates included age (years) and body mass index (BMI, kg/m^2^). Binary covariates involved gender (male or female), smoking or drinking status (never, smoker or drinker), marriage status (married or others), physical activity (yes or no), self-reported hypertension status (yes or no), residence type (rural area or urban community), and address region (northern or southern China, divided by the Qinling-Huaihe line). Multiple categorical covariates included education status (illiterate, elementary school, middle school, or above). To determine the final set of covariates, we performed a directed acyclic graph (DAG) analysis (eMethod 5).

### 2.5 Statistical Analyses

We used a semiparametric model with Cox proportional survival hazards regression to examine the risk estimates of ETEs and PM_2.5_ pollution on CMM. Initially, we developed a crude model, including ETEs, PM_2.5_, or single constituent, and the outcome. Subsequently, we further adjusted for DAG-selected covariates in the adjusted model. The weighted Schoenfeld residual test and variables’ variance inflation factors were successively conducted to examine proportional hazards assumption and potential multicollinearity, respectively. And we did not detect any violation. We performed the Akaike Information Criterion (AIC) to assess model fitness for the multidefined ETEs and determine optimal definitions. And those definitions with minimal AIC values were selected for further analyses. Stratified analyses were also conducted by several modifiers, involving age, gender, BMI status, marriage, education, residence type, regions, smoking, and hypertension. The Z-test was performed to determine the difference between subgroups. Besides, we performed the weighted quantile sum (WQS) regression model with logistic conjunction to examine the joint association of five highly correlated components with CMM (eMethod 6).^37,38^ Calculated risk estimates were exhibited with hazard ratios (HRs) and 95% confidence interval (CI) related to a specific increment for occurred-ETEs days or PM_2.5_ concentrations.

Additionally, we evaluated the interaction effects of ETEs and PM_2.5_ exposure on CMM (eMethod 6). We transformed PM_2.5_ pollution and its components into binary variables according to the 2021 interim target 1 from the WHO air quality guideline (annual average PM_2.5_: 35 μg/m^3^) or the median concentrations. Similarly, we converted HW11 and CS10 into binary variables based on their occurrence within the previous year. We then generated a categorical dummy variable with four levels, including nonheatwave and low-level PM_2.5_ (X_00_), non-HW and high-level PM_2.5_ (X_01_), HW and low-level PM_2.5_ (X_10_), and heatwave and high-level PM_2.5_ (X_11_). Similar dummy-variable classifications were also conducted for cold spell and PM_2.5_ components. The dummy variable was further included in the Cox regression model, with the X_00_ serving as reference. Afterward, we calculated relative excess risk due to interaction (RERI), attributable proportion due to interaction (AP), and the synergy index (S) for the additive interaction analysis, which was more informative into public health actions. While the corresponding 95%CI were calculated using the delta method.^39^

### 2.6 Sensitivity Analyses

First, we recalculated the time-varying exposure with 2-year time scale. Second, we eliminated those participants who had a follow-up duration of less than three years to assess the impact of follow-up time. Third, we omitted outcome events that occurred within the initial two years to minimize the risk of reverse causation. Fourth, we further adjusted for other confounders, including drinking status, physical exercise, sleep status, hypertension, and ambient ozone pollution upon the main model. Fifth, we also conducted a nested case-control study based on the current merge data set. Participants with CMM were included in the case group, while the control group was selected using the propensity score matching method with a matching ratio of 1:3 and a caliper value of 0.02 based on the selected factors, involving age, gender, BMI status (underweight, normal, overweight, and obesity), marital, education, and smoking status. We then performed a conditional logistic regression model to determine the association of PM_2.5_ and ETEs exposure with CMM. All the statistical analyses were operated in R 4.3.1, with statistical significance defined as a two-sided *p*-value less than 0.05.

## 3 Results

**Figure 1:**
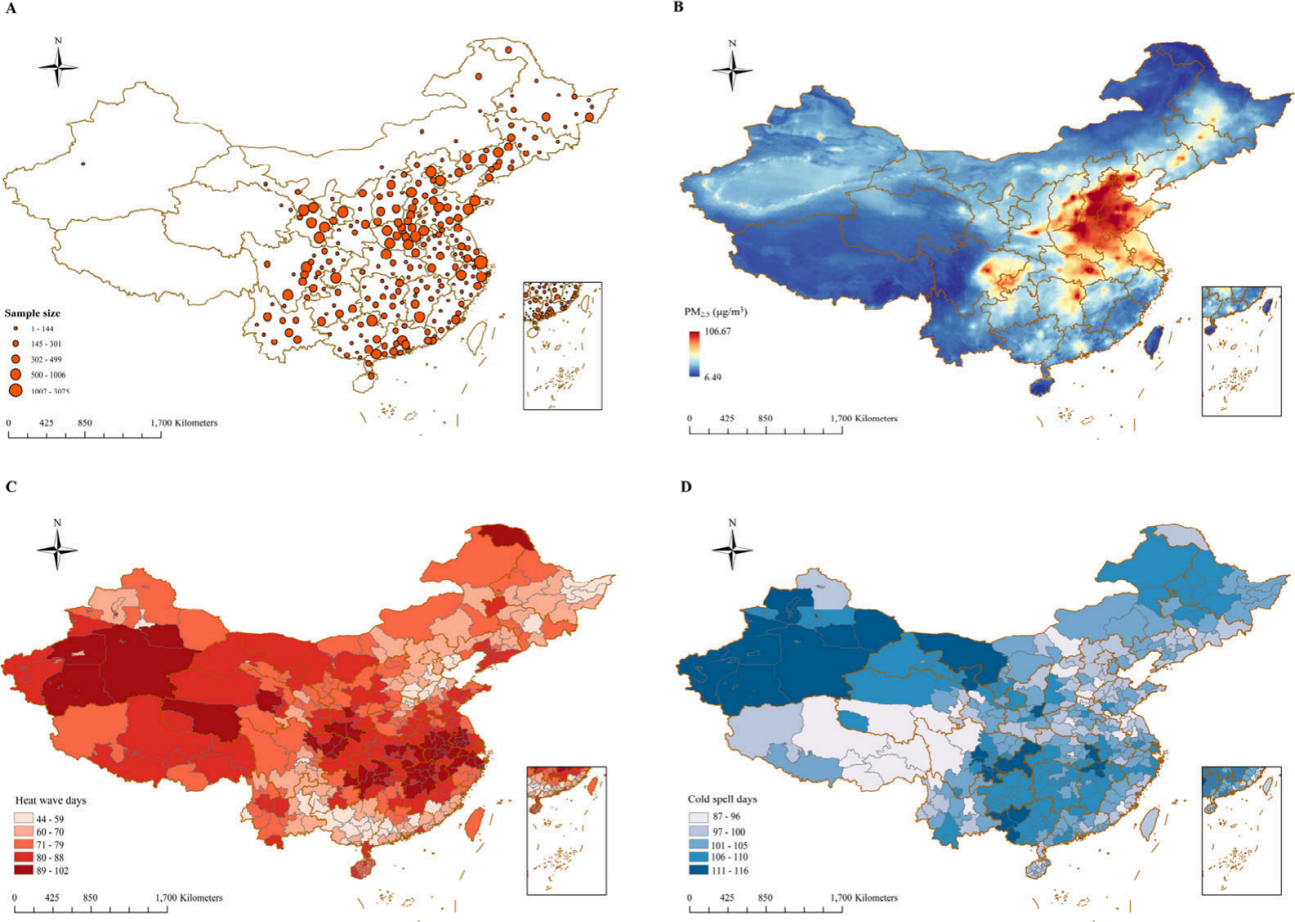
Spatial distribution of participants’ residential location, PM_2.5_ concentrations, and number of days of heatwave and cold spell during study periods. (A) Residential location; (B) PM_2.5_ concentrations; (C) heatwave, defined as 97.5th percentile threshold with a 3 consecutive days duration; and (D) cold spell, defined as 2.5th percentile threshold with 2 consecutive days duration.

We recruited a total of 64,140 participants with an average age of 51.89 years (SD = 20.58) in 258 Chinese cities between 2008 and 2020 (Figure 1A). The gender distribution was nearly equal, with 50.3% male participants. Approximately 53.2% of the participants were urbanites, and 47.5% were from northern China (Table 1). More detailed characteristics for each separate cohort can be found in Table S2. During the follow-up period of 0.4 million person-years, 1229 new-onset cases of CMM were finally identified, yielding an incidence rate of 3.07 per 1000 person-years. We observed significant differences between CMM (1.9%) and non-CMM (98.1%) groups concerning age, gender, BMI, marital status, education level, residence type, region, hypertension, smoking, and sleep. PM_2.5_ concentrations varied between 6.49 and 106.47 μg/m^3^ throughout the study duration, with higher levels in northern China (Figure 1B). The northwest region, particularly the Tarim Basin, experienced high levels of heatwave and cold spell (Figure 1). The lower-middle reaches of the Yangtze River were also affected by heatwave exposure (Figure 1C). Specifically, the annual PM_2.5_, HW11, and CS10 exposure for the participants were 40.65 (16.90) μg/m^3^, 5.55 (5.99), and 7.36 (6.29) days, respectively (Table 1).

**Table 1:** Baseline Characters and Environmental Exposure for the Study Participants^a^

Variables	Totally (*n* = 64140)	non-CMM (*n* = 62911)	CMM (*n* = 1229)	*p*-value
Demographic characters				
Age (years)	51.89 (20.58)	51.64 (20.63)	64.33 (12.21)	<0.001^b^
Gender (male)	32254 (50.3%)	31520 (50.1%)	734 (59.7%)	<0.001^b^
BMI (kg/m^2^)	22.15 (3.38)	22.12 (3.37)	23.74 (3.78)	<0.001^b^
Marital status (Married)	29298 (45.7%)	28625 (45.5%)	673 (54.8%)	<0.001^b^
Education				<0.001^b^
Illiteracy	18758 (29.2%)	18302 (29.1%)	456 (37.1%)	
Elementary school	17855 (27.8%)	17467 (27.8%)	388 (31.6%)	
Middle school or above	27527 (42.9%)	27142 (43.1%)	385 (31.3%)	
Urbanite	34106 (53.2%)	33507 (53.3%)	599 (48.7%)	<0.001^b^
Region (North)	30436 (47.5%)	29630 (47.1%)	806 (65.6%)	<0.001^b^
Health status				
Hypertension^c^	5173 (20.2%)	4841 (19.5%)	332 (38.4%)	<0.001^b^
Behavioral factors				
Smoker (former or current)	19154 (29.9%)	18874 (30%)	280 (22.8%)	<0.001^b^
Drinker (former or current)^d^	11225 (19.8%)	11012 (19.8%)	213 (20.6%)	0.525
Physical exercise^e^	20795 (33.2%)	20359 (33.1%)	436 (35.7%)	0.060
Enough sleep^f^	40128 (76.9%)	39464 (77.2%)	664 (65.7%)	<0.001^b^
Environmental factors				
PM_2.5_ (μg/m^3^)	40.65 (16.90)	40.49 (16.81)	49.35 (18.74)	<0.001^b^
NO_3_^–^ (μg/m^3^)	1.94 (0.70)	1.93 (0.70)	2.30 (0.78)	<0.001^b^
SO_4_^2–^ (μg/m^3^)	7.51 (3.05)	7.49 (3.04)	8.95 (3.37)	<0.001^b^
NH_4_^+^ (μg/m^3^)	6.08 (2.71)	6.05 (2.70)	7.36 (2.90)	<0.001^b^
OM (μg/m^3^)	9.89 (3.62)	9.85 (3.60)	11.76 (4.16)	<0.001^b^
BC (μg/m^3^)	1.94 (0.70)	1.93 (0.70)	2.30 (0.78)	<0.001^b^
HW11 (days)	5.55 (5.99)	5.52 (5.98)	7.08 (6.10)	<0.001^b^
CS10 (days)	7.36 (6.29)	7.33 (6.30)	9.11 (5.52)	<0.001^b^

a Abbreviations: CMM, cardiometabolic multimorbidity; BMI, body mass index; PM_2.5_, fine particulate matter; NO_3_^–^, nitrate; SO_4_^2–^, sulfate; NH_4_^+^, ammonium; OM, organic matter; BC, black carbon; HW11, heatwave frequency for the definition of daily average temperature equal to or higher than 97.5th percentile for at least 3 consecutive days; CS10, cold spell frequency for the definition of daily average temperature lower than 2.5th percentile with at least 2 consecutive days.

b Z-test *p*-value < 0.05.

c 38440 missing data.

d 7368 missing data.

e 1479 missing data.

f 11986 missing data.

**Figure 2:**
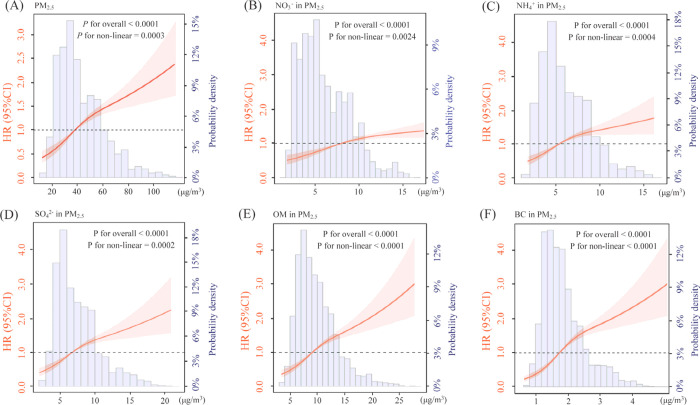
Exposure-response curve of PM_2.5_ with CMM occurrence. (A) PM_2.5_, (B) NO_3_^–^, (C) NH_4_^+^, (D) SO_4_^2–^, (E) OM, and (F) BC. Abbreviations: CMM, cardiometabolic multimorbidity; HR, hazard ratio; CI, confidence interval; PM_2.5_, fine particulate matter; NO_3_^–^, nitrate; SO_4_^2–^, sulfate; NH_4_^+^, ammonium; OM, organic matter; and BC, black carbon. Note: The regression models were fully adjusted for age, gender, body mass index, marital status, education, smoking status, residence type, and region.

We observed near-linear and positive associations between PM_2.5_ with its constituents and CMM occurrence (Figure 2). In the linear analyses, elevated risks of CMM were associated with PM_2.5_ and its constituents, with estimated HRs of 1.179 for PM_2.5_ (1.139–1.220, per 10 μg/m^3^), 1.047 for NO_3_^–^ (1.033–1.062, 1 μg/m^3^), 1.092 for SO_4_^2–^ (1.071–1.113, 1 μg/m^3^), 1.089 for NH_4_^+^ (1.065–1.113, 1 μg/m^3^), 1.088 for OM (1.071–1.104, 1 μg/m^3^), and 1.054 for BC (1.045–1.062, 0.1 μg/m^3^) (Table 2). A mixture analysis also revealed a positive association between the combined exposure to 5 constituents and CMM, with an HR of 1.160 (1.127–1.195). The weights of BC (62.4%) and SO_4_^2–^ (33.7%) predominated in the mixed exposure (Figure S5), indicating the major contributions to CMM. Further stratification analysis showed that marital status, residence, and region type were the modifiers for the above associations (Tables 3 and S4). Specifically, the participants without cohabitants (HR = 1.296, 1.232–1.364), rural residents (1.238, 1.179–1.299), and southerners (1.356, 1.269–1.449) suffered from greater risk of CMM associated with PM_2.5_ pollution than the counterparts. Similar modification effects were observed for NO_3_^–^, NH_4_^+^, and SO_4_^2–^ exposure. Additionally, we found that overweight individuals had a higher estimated risk of developing CMM when exposed to OM (1.113, 1.085–1.143) and BC (1.068, 1.053–1.084) compared to the normal-weight participants.

**Table 2:** Associations between PM_2.5_ with Its Constituents and CMM^a^

	Crude model	Main model
PM_2.5_	1.499 (1.456, 1.544)	1.179 (1.139, 1.220)
NO_3_^–^	1.146 (1.132, 1.160)	1.047 (1.033, 1.062)
SO_4_^2–^	1.251 (1.230, 1.272)	1.092 (1.071, 1.113)
NH_4_^+^	1.266 (1.242, 1.290)	1.089 (1.065, 1.113)
OM	1.212 (1.196, 1.227)	1.088 (1.071, 1.104)
BC	1.116 (1.109, 1.124)	1.054 (1.045, 1.062)

a Abbreviations: CMM, cardiometabolic multimorbidity; HR, hazard ratio; CI, confidence intervals; PM_2.5_, fine particulate matter; NO_3_^–^, nitrate; SO_4_^2–^, sulfate; NH_4_^+^, ammonium; OM, organic matter; and BC, black carbon. Note: Main model was fully adjusted for age, gender, body mass index, marital status, education, smoking status, residence type, and region. HRs for CMM were estimated by a specific increase for PM_2.5_ (10 μg/m^3^), NO_3_^–^ (1 μg/m^3^), SO_4_^2–^ (1 μg/m^3^), NH_4_^+^ (1 μg/m^3^), OM (1 μg/m^3^), and BC (0.1 μg/m^3^).

**Table 3:** Stratification Analysis for the Association of PM_2.5_ and Extreme Temperature Events with CMM^a^

	PM_2.5_		HW11		CS10	
	HR (95%CI)	*p*-Z_test_	HR (95%CI)	*p*-Z_test_	HR (95%CI)	*p*-Z_test_
Gender						
Male	1.157 (1.096, 1.221)		1.011 (0.996, 1.025)		1.082 (1.065, 1.099)	
Female	1.202 (1.149, 1.258)	0.286	1.025 (1.014, 1.037)	0.118	1.096 (1.082, 1.110)	0.226
Age, years						
<45	1.143 (0.887, 1.473)		1.054 (0.999, 1.111)		1.138 (1.081, 1.197)	
45–65	1.431 (1.344, 1.523)	0.092	1.018 (1.003, 1.034)	0.223	1.067 (1.050, 1.084)	0.019^b^
≥65	1.157 (1.109, 1.207)	0.928	1.015 (1.003, 1.027)	0.172	1.081 (1.065, 1.097)	0.058
BMI, kg/m^2^						
Underweight	1.224 (1.082, 1.385)	0.362	1.035 (1.006, 1.064)	0.381	1.104 (1.063, 1.146)	0.812
Normal	1.151 (1.100, 1.205)		1.021 (1.009, 1.032)		1.099 (1.084, 1.113)	
Overweight	1.227 (1.156, 1.302)	0.096	1.008 (0.991, 1.025)	0.208	1.068 (1.049, 1.088)	0.014^b^
Education level						
Illiteracy	1.245 (1.176, 1.319)		1.025 (1.010, 1.039)		1.083 (1.066, 1.101)	
Element	1.231 (1.157, 1.308)	0.778	1.006 (0.990, 1.023)	0.100	1.069 (1.049, 1.090)	0.314
Middle or above	1.193 (1.118, 1.273)	0.331	1.030 (1.014, 1.047)	0.619	1.103 (1.083, 1.124)	0.134
Smoke status						
Yes	1.187 (1.103, 1.277)		0.997 (0.977, 1.017)		1.075 (1.052, 1.098)	
No	1.178 (1.133, 1.225)	0.864	1.025 (1.015, 1.035)	0.016^b^	1.096 (1.084, 1.109)	0.105
Marital status						
Married	1.106 (1.056, 1.159)		1.000 (0.987, 1.013)		1.052 (1.036, 1.069)	
Others^c^	1.296 (1.232, 1.364)	<0.001^b^	1.036 (1.023, 1.049)	<0.001^b^	1.111 (1.096, 1.126)	<0.001^b^
Residence type						
Urban	1.136 (1.080, 1.194)		1.015 (1.003, 1.028)		1.111 (1.095, 1.127)	
Rural	1.238 (1.179, 1.299)	0.016^b^	1.022 (1.009, 1.035)	0.484	1.071 (1.055, 1.086)	<0.001^b^
Region type						
North	1.125 (1.080, 1.172)		1.025 (1.013, 1.037)		1.098 (1.084, 1.111)	
South	1.356 (1.269, 1.449)	<0.001^b^	1.014 (1.000, 1.029)	0.273	1.081 (1.062, 1.100)	0.167

a Abbreviations: PM_2.5_, fine particulate matter; CMM, cardiometabolic multimorbidity; HR, hazard ratio; 95%CI, 95% confidence intervals; HW11, heat wave frequency for the definition of daily average temperature equal to or higher than 97.5th percentile for at least 3 consecutive days; CS10, cold spell frequency for the definition of daily average temperature lower than 2.5th percentile with at least 2 consecutive days; BMI, body mass index.

b Note: Z-test *p*-value < 0.05, suggesting that the differences between subgroups are statistically significant. Each stratification controlled for all factors (age, gender, BMI, marital status, education level, smoking status, residence, and region type) except the stratification factor itself.

c Others included unmarried, separated, divorced or widowed.

**Figure 3:**
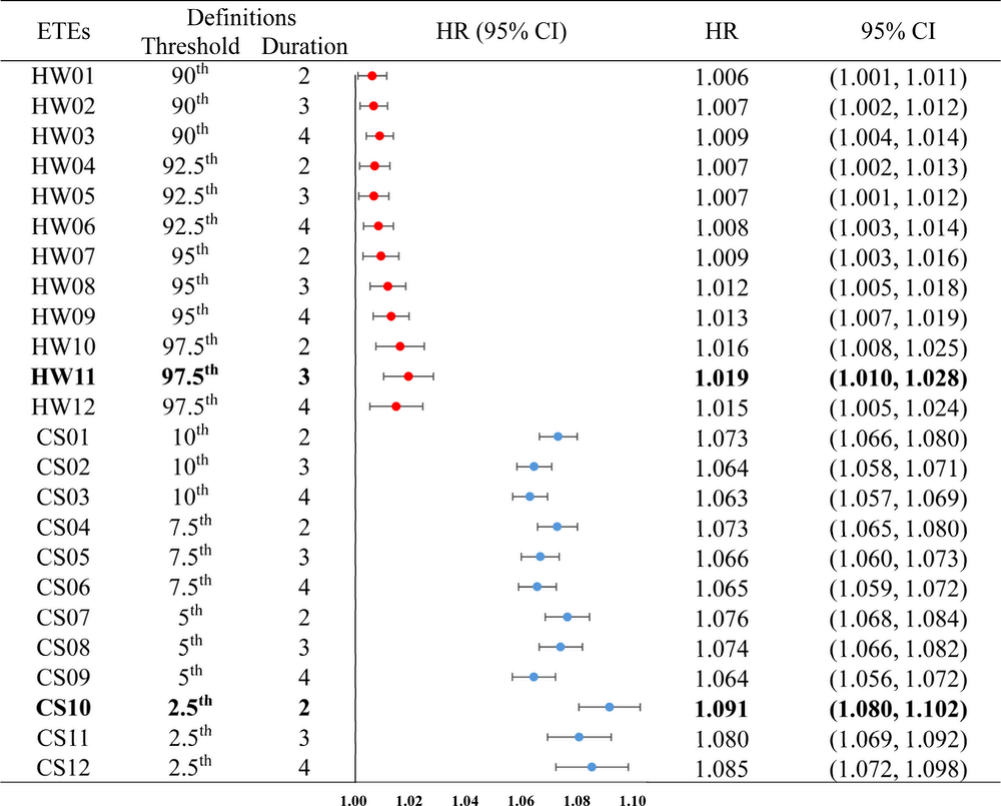
Associations between different definitions of ETEs and cardiometabolic multimorbidity occurrence. ETEs, extreme temperature events; HR, hazard ratio; CI, confidence interval; HW, heatwave; CS, cold spell. Notes: bolded type indicates that the AIC value was minimum under this definition and also selected for subsequent analysis.

Figure 3 presented the associations of ETEs with CMM under different definitions. We found that both heatwave and cold spell were positively associated with CMM occurrence after fully adjusting for covariates. Interestingly, the risk estimates of cold spell appeared to be stronger than heatwave exposure. The estimated HR values ranged from 1.063 to 1.091 for cold spell, with the largest HR of 1.091 (1.080–1.102) observed in the CS10. For the heatwave, HW11 showed the largest HRs of 1.019 (1.010–1.028) and minimum AIC value for CMM incidents. Stratification analysis summarized that the associations between heatwave and increased CMM risk were stronger among nonsmokers, while cold spell contributed to greater risks among the urbanites (Table 3). Besides, higher risk estimates for the above association were recognized among participants without cohabitants, with HRs of 1.036 (1.023–1.049) for HW11 exposure and 1.111 (1.096–1.126) for CS10 exposure, respectively (Table 3). Additionally, the normal-weight and the younger adults (<45 years) suffered greater cold-related risks of CMM.

**Figure 4:**
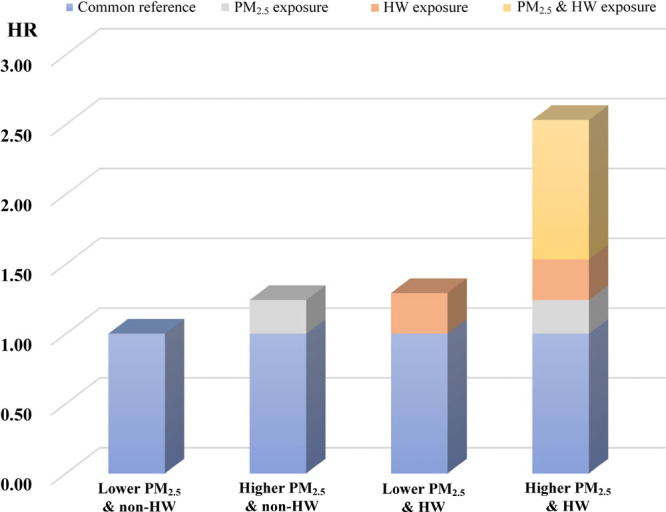
Relative risk with contributions of CMM occurrence from different exposure categories. Higher PM_2.5_ means heavier pollution over 35 μg/m^3^, and HW is defined as 97.5th percentile threshold with 3 consecutive days duration. HR, hazard ratio; HW, heatwave.

We observed additive interactive effects for coexposure to heatwave and PM_2.5_ with its constituents on CMM (Table 4). For HW11 and PM_2.5_, the HR_01_, HR_10_, and HR_11_ for CMM incidents were 1.241 (0.972–1.586), 1.292 (1.021–1.636), and 2.532 (2.037–3.147), respectively (Figure 4). A significant synergistic effect for the above association was observed with the RERI, AP, and S was 0.999 (0.663–1.334), 0.394 (0.256–0.529), and 2.873 (1.325–6.230), respectively. Similarly, we also found significant synergistic effects of exposure to heatwave and PM_2.5_ constituents on CMM, indicated by RERI > 0, AP > 0, S > 1, and all *p*-values < 0.05. However, we did not observe significant interactions between cold spell and PM_2.5_ with its 5 constituents on CMM.

**Table 4:** Additive Interaction Effects of Exposure to Extreme Temperature Events and PM_2.5_ on CMM^a^

	RERI	AP	S
HW11			
PM_2.5_	0.999 (0.663, 1.334)	0.394 (0.256, 0.529)	2.873 (1.325, 6.230)
NO_3_^–^	0.794 (0.500, 1.089)	0.375 (0.220, 0.520)	3.469 (1.062, 11.327)
NH_4_^+^	0.838 (0.527, 1.149)	0.375 (0.233, 0.516)	3.098 (1.189, 8.070)
BC	0.815 (0.322, 1.308)	0.228 (0.093, 0.364)	1.465 (1.103, 1.945)
OM	1.003 (0.659, 1.346)	0.394 (0.263, 0.526)	2.856 (1.383, 5.897)
SO_4_^2–^	0.845 (0.484, 1.207)	0.321 (0.184, 0.457)	2.068 (1.243, 3.439)
CS10			
PM_2.5_	0.692 (−0.692, 2.092)	0.066 (−0.064, 0.120)	1.078 (0.924, 1.258)
NO_3_^–^	0.284 (−0.775, 1.342)	0.036 (−0.098, 0.170)	1.043 (0.889, 1.224)
NH_4_^+^	0.199 (−0.973, 1.371)	0.023 (−0.113, 0.159)	1.027 (0.877, 1.202)
BC	0.695 (−1.043, 2.432)	0.055 (−0.080, 0.190)	1.063 (0.910, 1.243)
OM	0.108 (−1.226, 1.441)	0.011 (−0.128, 0.150)	1.013 (0.866, 1.185)
SO_4_^2–^	0.359 (−0.880, 1.597)	0.040 (−0.096, 0.175)	1.047 (0.892, 1.228)

a Abbreviations: PM_2.5_, fine particulate matter; CMM, cardiometabolic multimorbidity; RERI, relative excess risk due to interaction; AP, attributable proportion due to interaction; S, synergy index; HW11, heat wave frequency for the definition of daily average temperature equal to or higher than 97.5th percentile for at least 3 consecutive days; CS10, cold spell frequency for the definition of daily average temperature lower than 2.5th percentile with at least 2 consecutive days; NO_3_^–^, nitrate; SO_4_^2–^, sulfate; NH_4_^+^, ammonium; OM, organic matter; and BC, black carbon.

Series of sensitivity analyses showed our findings’ robustness. First, we examined the associations of CMM incidents with PM_2.5_ constituents and ETEs on a 2-year time scale. We also observed significantly positive associations (Table S6). We then excluded the participants with a follow-up of less than 3 years or those who developed CMM within 2 years, respectively. And the results were generally consistent with the main analysis (Tables S7 and S8). We further adjusted for drinking status, exercise habits, sleep status, and ozone concentrations in the main model, and the association remained significant (Table S9). Furthermore, we developed a nested case-control study including 1229 new CMM cases and 3687 free-CMM participants as control. After adjusting several DAG-selected covariates, we observed positive associations of CMM with PM_2.5_, heatwave, and cold spell (Table S10). All the above analyses indicated our result’s robustness.

## 4 Discussion

To the best of our knowledge, this is the first large-scale cohort study exploring the independent and interaction effects of exposure to ETEs and PM_2.5_ with constituents on CMM incidence worldwide. Our findings indicate that increased levels of PM_2.5_ pollution, as well as heatwave and cold spell, are environmental risk factors associated with the occurrence of CMM. These results remain consistent across 5 kinds of PM_2.5_ constituents, with BC and SO_4_^2–^ playing a particularly significant role. Furthermore, we found synergistically detrimental effects resulting from the coexposure to PM_2.5_ and heatwave.

Previous studies conducted in China and the UK have examined the association between PM_2.5_ pollution and CMM morbidity.^7,14,15^ The UREP survey, a cross-sectional study involving 222,179 Chinese adults, indicated that a 10 μg/m^3^ increase in PM_2.5_ concentrations is related to a 2.2–7.6% higher risk of CMM.^7^ Another two cohort studies, enrolling about 0.4 million UK Biobank participants free of CMDs, also reported positive association between PM_2.5_ pollution and the progression trajectory of CMM.^14,15^ Our results were consistent with the above findings. Discrepancies in estimated risk values may be attributed to variations in participant demographics, outcome definitions, PM_2.5_ concentrations, and chemical constituents.^16,40^ Furthermore, we observed significant associations between 5 major PM_2.5_ constituents (NO_3_^–^, OM, SO_4_^2–^, NH_4_^+^, and BC) and higher risks of CMM. To our knowledge, there was no existing direct evidence on PM_2.5_ constituents and CMM. Published studies on single CMDs might support our findings.^40−42^ The CFPS study revealed the positive associations for the 4 constituents (NO_3_^–^, SO_4_^2–^, NH_4_^+^, and BC) exposure and cardiovascular disease, indicated by calculated HRs of 1.294 to 1.721.^40^ Evidence from the Jinchang cohort reported that long-term exposure to NO_3_^–^, NH_4_^+^, OM, and BC were significantly associated with diabetes.^41^ Particularly, BC emerged as the primary contributor in CMM development. BC in PM_2.5_ primarily stems from the incomplete combustion of fossil fuels and biomass.^43^ Our results suggest that controlling the use of fossil fuels and developing cleaner fuels may be helpful for preventing CMM.

Another main finding was the positive association between ETEs and CMM occurrence. Over the past decades, emerging studies have evaluated the effects of extreme temperatures on single CMDs.^44−46^ For instance, a prior study in 31 Chinese capital cities reported that cold spell (CS02) was associated with an increased risk for IHD (RR = 1.67, 1.45–1.89), stroke (1.52, 1.35–1.69), and diabetes (1.66, 1.43–1.89).^45^ Similar associations were also observed with heatwave (HW05).^46^ However, findings from the U.S. reported that there were null associations between extreme heat exposure (99th percentile) and cardiovascular disease or diabetes.^44^ The heterogeneous results observed in these studies may be attributed to variations in participant characteristics, lifestyle habits, regional climate patterns, definitions of ETEs, and study designs.^24,47^ However, the above evidence primarily focuses on the acute effect of ETEs, which might be insufficient for understanding the development of chronic diseases such as CMM. Consequently, our study focused on chronic effects by calculating the days of heatwave or cold spell over the past year.^48^

With regard to the underlying mechanisms of exposure to PM_2.5_ and ETEs on CMM, there was a lack of clear explanations yet. Several generally accepted views were that PM_2.5_ pollution could trigger systematic oxidative stress and inflammation, induce intestinal microbial dysbiosis, accelerate atherosclerosis, and disrupt cardiac autonomic function,^49−51^ wherein various PM_2.5_ constituents exert important roles. BC could induce excess reactive oxygen species and inflammatory markers, as well as alter DNA methylation, thereby accelerating cardiometabolic impairment.^52,53^ Available biomechanisms for the heatwave involved triggering autonomic neuropathy, accelerating cellular electrolyte disorders, or interfering with glucose tolerance.^46,54^ The possible mechanistic impact of the cold spell may be due to an increased plasma fibrinogen and coagulation factor VII, potentially damaging vascular endothelium and triggering thrombosis.^45,55^ Additionally, the cold may also activate sympathetic nervous system and renin-angiotensin system, leading to higher heart rate and increased peripheral vascular resistance.^56^ Nonetheless, the exact mechanism is worth elucidating in future studies.

Additionally, our findings provide novel evidence suggesting a synergistic effect of air pollution and ETEs on human health. Several epidemiological studies have reported similar interaction effects on mortality or hospital visits.^24,25,57^ However, the possible synergistic effects of PM_2.5_ pollution and ETEs exposure on CMM are not assessed yet. Currently, we evaluated the additive interactions of ETEs and PM_2.5_ pollution on CMM development. A significantly synergistic effect was observed with coexposure to heatwave (HW11) and heavier PM_2.5_ pollution (>35 μg/m^3^). In line with our findings, a muti-center study across 24 countries reported that heat-related cardiovascular mortality increased by 2.07 (2.04–2.10), 4.29 (4.26–4.32), and 7.33 (7.29–7.37) under low, median, and high PM_2.5_ levels, respectively, suggesting a potential interaction effect.^58^ Another finding from the CLHLS cohort also reported such a synergistic adverse effect of heatwave and PM_2.5_ pollution on hypertension incidence.^59^ Several existing biomechanisms could potentially explain our findings. A plausible view indicated that high temperatures could increase the uptake of PM_2.5_ by triggering an elevated blood flow rate, skin permeability, and respiratory rate.^23^ Besides, previous evidence showed heatwave and PM_2.5_ pollution share common biological pathways involving accelerated oxidative stress injury and systemic inflammation,^23,60,61^ which may provoke synergetic health effects. However, further studies are needed to elucidate the specific mechanisms for the current synergetic interaction.

Our subgroup analyses revealed several modifiers in the association of PM_2.5_ pollution and ETEs with CMM morbidity. First, we found that the singles were more susceptible to PM_2.5_ and ETEs exposure on CMM. From the sociologic viewpoint, the married commonly have well-established social support systems and also have more accessibility to emotional assistance via the marital relationship,^62,63^ which may help to alleviate stresses from air pollution and ETEs. Besides, the singles are more likely to coexist with CMM-related unhealthy lifestyles, such as smoking or excessive drinking.^64^ Second, there were stronger risk estimates for PM_2.5_ and cold spell exposure among rural and urban residents, respectively. For the PM_2.5_ pollution, two published studies were in line with our findings.^7,65^ And the plausible reasons might attribute to the higher frequency of solid fuels usage in rural cooking and heating,^65^ which was reported to have a synergistic effect with PM_2.5_ pollution. In addition, the urban residents typically suffer from the cold faster and longer during a cold spell due to the urban heat island.^66^ Third, we observed that the risk estimate of PM_2.5_ pollution was greater in southern China. We hypothesized that the main reason might be the higher PM_2.5_ infiltration and exposure factors in southern than northern China.^67,68^ Specifically, residents in the southern regions open windows for ventilation more frequently than those in the north, enabling a higher infiltration of ambient PM_2.5_ indoors and elevating actual human exposure.^67^

The strengths of this study mainly involved the perspective, large sample size, and nationally representative cohorts with long-term follow-up visits. Specifically, participants were recruited from 258 Chinese cities, covering more than 6 different climatic zones, which greatly enhanced our findings’ generalizability. The large sample size, consisting of 64,140 participants across a wide age range, ensured sufficient statistical power for analyzing joint effects and modification effects. In addition, our study focused on the interaction effects of PM_2.5_ pollution and ETEs exposure, which extended our understanding of health impacts of the atmospheric environment.

A number of limitations should also be acknowledged. First, the information on CMM was obtained through self-reported questionnaires during each follow-up survey. This approach may underestimate the actual risk effects as self-reported morbidity tends to be lower than the true incidence. Second, PM_2.5_ and its constituents’ concentrations were measured at the city level for current risk analysis due to the unavailability of exact residential addresses. Thus, misclassification bias was inevitable for current exposure measurement. However, published studies suggested that such a bias is unlikely to alter the current exposure-response relationship greatly.^69^ Third, several confounders, the usages of air conditioning or heating systems, were not adjusted in the current associations for a lack of relevant information. Fourth, we excluded those participants with CMDs in the baseline survey. Among these participants, there may be a higher proportion of susceptible people with earlier developed CMM, which may underestimate the risk effects. Fifth, the four included cohorts overlapped somewhat in terms of city coverage and follow-up periods and lacked the weights of populations, which may hamper the representativeness of our findings. Finally, estimates of personal exposure relied on the number of days of ETEs, focusing mainly on the frequency of heatwave or cold spell. A comprehensive indicator that considers both the intensity and frequency of extreme temperature events would be beneficial to develop and implement in future studies.

Generally, our study provided epidemiological evidence that exposure to heatwave, cold spell, and PM_2.5_ was independently associated with an increased risk of CMM among Chinese adults. Moreover, we observed a synergistic interaction between heatwave and PM_2.5_ and its constituents in triggering CMM occurrence. Under the climate change scenario, our novel findings emphasized the multiple benefits of mitigating air pollution to promote cardiometabolic health.

## Supporting Information Available

Supplemental eMethods, definitions of extreme temperature events (Table S1), baseline characteristics for the four cohorts’ participants (Table S2), the Spearman correlation matrix between PM_2.5_ with its constituents’ concentrations (Table S3), stratification analysis (Table S4), Akaike information criterion values (Table S5), sensitivity analyses (Tables S6–S10), study flowchart (Figure S1), the geographic distribution of NCEI monitoring stations (Figure S2), directed acyclic graph (Figure S3), the participants age distribution (Figure S4), and weights of 5-kinds PM_2.5_ constituents mixture on CMM (Figure S5) (DOCX)

## References

[ref1] Han Y.; Hu Y.; Yu C.; Guo Y.; Pei P.; Yang L.; Chen Y.; Du H.; Sun D.; Pang Y.; Chen N.; Clarke R.; Chen J.; Chen Z.; Li L.; Lv J. Lifestyle, cardiometabolic disease, and multimorbidity in a prospective Chinese study. Eur. Heart J. 2021, 42 (34), 3374–3384. 10.1093/eurheartj/ehab413. 34333624 PMC8423468

[ref2] Valderas J. M.; Starfield B.; Sibbald B.; Salisbury C.; Roland M. Defining comorbidity: implications for understanding health and health services. Ann. Fam Med. 2009, 7 (4), 357–63. 10.1370/afm.983. 19597174 PMC2713155

[ref3] GBD 2019 Diseases and Injuries Collaborators. GBD, Global burden of 369 diseases and injuries in 204 countries and territories, 1990–2019: a systematic analysis for the Global Burden of Disease Study 2019. Lancet 2020, 396 ( (10258), ), 1204–1222. 10.1016/S0140-6736(20)30925-9 33069326 PMC7567026

[ref4] Di Angelantonio E.; Kaptoge S.; Wormser D.; Willeit P.; Butterworth A. S.; Bansal N.; O’Keeffe L. M.; Gao P.; Wood A. M.; Burgess S.; Freitag D. F.; Pennells L.; Peters S. A.; Hart C. L.; Håheim L. L.; Gillum R. F.; Nordestgaard B. G.; Psaty B. M.; Yeap B. B.; Knuiman M. W.; Nietert P. J.; Kauhanen J.; Salonen J. T.; Kuller L. H.; Simons L. A.; van der Schouw Y. T.; Barrett-Connor E.; Selmer R.; Crespo C. J.; Rodriguez B.; Verschuren W. M.; Salomaa V.; Svärdsudd K.; van der Harst P.; Björkelund C.; Wilhelmsen L.; Wallace R. B.; Brenner H.; Amouyel P.; Barr E. L.; Iso H.; Onat A.; Trevisan M.; D’Agostino R. B. Sr; Cooper C.; Kavousi M.; Welin L.; Roussel R.; Hu F. B.; Sato S.; Davidson K. W.; Howard B. V.; Leening M. J.; Leening M.; Rosengren A.; Dörr M.; Deeg D. J.; Kiechl S.; Stehouwer C. D.; Nissinen A.; Giampaoli S.; Donfrancesco C.; Kromhout D.; Price J. F.; Peters A.; Meade T. W.; Casiglia E.; Lawlor D. A.; Gallacher J.; Nagel D.; Franco O. H.; Assmann G.; Dagenais G. R.; Jukema J. W.; Sundström J.; Woodward M.; Brunner E. J.; Khaw K. T.; Wareham N. J.; Whitsel E. A.; Njølstad I.; Hedblad B.; Wassertheil-Smoller S.; Engström G.; Rosamond W. D.; Selvin E.; Sattar N.; Thompson S. G.; Danesh J. Association of Cardiometabolic Multimorbidity With Mortality. JAMA 2015, 314 (1), 52–60. 10.1001/jama.2015.7008. 26151266 PMC4664176

[ref5] Canoy D.; Tran J.; Zottoli M.; Ramakrishnan R.; Hassaine A.; Rao S.; Li Y.; Salimi-Khorshidi G.; Norton R.; Rahimi K. Association between cardiometabolic disease multimorbidity and all-cause mortality in 2 million women and men registered in UK general practices. BMC Med. 2021, 19 (1), 258 10.1186/s12916-021-02126-x. 34706724 PMC8555122

[ref6] Zhang D.; Tang X.; Shen P.; Si Y.; Liu X.; Xu Z.; Wu J.; Zhang J.; Lu P.; Lin H.; Gao P. Multimorbidity of cardiometabolic diseases: prevalence and risk for mortality from one million Chinese adults in a longitudinal cohort study. BMJ. Open 2019, 9 (3), e024476 10.1136/bmjopen-2018-024476. PMC6443196 30833320

[ref7] Su B.; Liu C.; Chen L.; Wu Y.; Li J.; Zheng X. Long-term exposure to PM_2.5_ and O_3_ with cardiometabolic multimorbidity: Evidence among Chinese elderly population from 462 cities. Ecotoxicol Environ. Saf 2023, 255, 114790 10.1016/j.ecoenv.2023.114790. 36948004

[ref8] Adair L. S.; Gordon-Larsen P.; Du S. F.; Zhang B.; Popkin B. M. The emergence of cardiometabolic disease risk in Chinese children and adults: consequences of changes in diet, physical activity and obesity. Obes Rev. 2014, 15 (S1), 49–59. 10.1111/obr.12123. 24341758 PMC3947601

[ref9] Chudasama Y. V.; Khunti K.; Gillies C. L.; Dhalwani N. N.; Davies M. J.; Yates T.; Zaccardi F. Healthy lifestyle and life expectancy in people with multimorbidity in the UK Biobank: A longitudinal cohort study. PLoS Med. 2020, 17 (9), e1003332 10.1371/journal.pmed.1003332. 32960883 PMC7508366

[ref10] Freisling H.; Viallon V.; Lennon H.; Bagnardi V.; Ricci C.; Butterworth A. S.; Sweeting M.; Muller D.; Romieu I.; Bazelle P.; Kvaskoff M.; Arveux P.; Severi G.; Bamia C.; Kühn T.; Kaaks R.; Bergmann M.; Boeing H.; Tjønneland A.; Olsen A.; Overvad K.; Dahm C. C.; Menéndez V.; Agudo A.; Sánchez M. J.; Amiano P.; Santiuste C.; Gurrea A. B.; Tong T. Y. N.; Schmidt J. A.; Tzoulaki I.; Tsilidis K. K.; Ward H.; Palli D.; Agnoli C.; Tumino R.; Ricceri F.; Panico S.; Picavet H. S. J.; Bakker M.; Monninkhof E.; Nilsson P.; Manjer J.; Rolandsson O.; Thysell E.; Weiderpass E.; Jenab M.; Riboli E.; Vineis P.; Danesh J.; Wareham N. J.; Gunter M. J.; Ferrari P. Lifestyle factors and risk of multimorbidity of cancer and cardiometabolic diseases: a multinational cohort study. BMC Med. 2020, 18 (1), 5 10.1186/s12916-019-1474-7. 31918762 PMC6953215

[ref11] Liu F.; Chen G.; Huo W.; Wang C.; Liu S.; Li N.; Mao S.; Hou Y.; Lu Y.; Xiang H. Associations between long-term exposure to ambient air pollution and risk of type 2 diabetes mellitus: A systematic review and meta-analysis. Environ. Pollut. 2019, 252, 1235–1245. 10.1016/j.envpol.2019.06.033. 31252121

[ref12] Wolf K.; Hoffmann B.; Andersen Z. J.; Atkinson R. W.; Bauwelinck M.; Bellander T.; Brandt J.; Brunekreef B.; Cesaroni G.; Chen J.; de Faire U.; de Hoogh K.; Fecht D.; Forastiere F.; Gulliver J.; Hertel O.; Hvidtfeldt U. A.; Janssen N. A. H.; Jørgensen J. T.; Katsouyanni K.; Ketzel M.; Klompmaker J. O.; Lager A.; Liu S.; MacDonald C. J.; Magnusson P. K. E.; Mehta A. J.; Nagel G.; Oftedal B.; Pedersen N. L.; Pershagen G.; Raaschou-Nielsen O.; Renzi M.; Rizzuto D.; Rodopoulou S.; Samoli E.; van der Schouw Y. T.; Schramm S.; Schwarze P.; Sigsgaard T.; Sørensen M.; Stafoggia M.; Strak M.; Tjønneland A.; Verschuren W. M. M.; Vienneau D.; Weinmayr G.; Hoek G.; Peters A.; Ljungman P. L. S. Long-term exposure to low-level ambient air pollution and incidence of stroke and coronary heart disease: a pooled analysis of six European cohorts within the ELAPSE project. Lancet Planet Health 2021, 5 (9), e620–e632. 10.1016/S2542-5196(21)00195-9. 34508683

[ref13] Sang S.; Chu C.; Zhang T.; Chen H.; Yang X. The global burden of disease attributable to ambient fine particulate matter in 204 countries and territories, 1990–2019: A systematic analysis of the Global Burden of Disease Study 2019. Ecotoxicol Environ. Saf 2022, 238, 113588 10.1016/j.ecoenv.2022.113588. 35525115

[ref14] Luo H.; Zhang Q.; Yu K.; Meng X.; Kan H.; Chen R. Long-term exposure to ambient air pollution is a risk factor for trajectory of cardiometabolic multimorbidity: A prospective study in the UK Biobank. EBioMedicine 2022, 84, 104282 10.1016/j.ebiom.2022.104282. 36174399 PMC9520206

[ref15] Zou H.; Zhang S.; Cai M.; Qian Z. M.; Zhang Z.; Chen L.; Wang X.; Arnold L. D.; Howard S. W.; Li H.; Lin H. Ambient air pollution associated with incidence and progression trajectory of cardiometabolic diseases: A multi-state analysis of a prospective cohort. Sci. Total Environ. 2023, 862, 160803 10.1016/j.scitotenv.2022.160803. 36493826

[ref16] Yang Y.; Ruan Z.; Wang X.; Yang Y.; Mason T. G.; Lin H.; Tian L. Short-term and long-term exposures to fine particulate matter constituents and health: A systematic review and meta-analysis. Environ. Pollut. 2019, 247, 874–882. 10.1016/j.envpol.2018.12.060. 30731313

[ref17] Geng G. N.; Zhang Q.; Tong D.; Li M.; Zheng Y. X.; Wang S. W.; He K. B. Chemical composition of ambient PM_2.5_ over China and relationship to precursor emissions during 2005–2012. Atmospheric Chemistry and Physics 2017, 17 (14), 9187–9203. 10.5194/acp-17-9187-2017.

[ref18] Alahmad B.; Khraishah H.; Royé D.; Vicedo-Cabrera A. M.; Guo Y.; Papatheodorou S. I.; Achilleos S.; Acquaotta F.; Armstrong B.; Bell M. L.; Pan S. C.; de Sousa Zanotti Stagliorio Coelho M.; Colistro V.; Dang T. N.; Van Dung D.; De’ Donato F. K.; Entezari A.; Guo Y. L.; Hashizume M.; Honda Y.; Indermitte E.; Íñiguez C.; Jaakkola J. J. K.; Kim H.; Lavigne E.; Lee W.; Li S.; Madureira J.; Mayvaneh F.; Orru H.; Overcenco A.; Ragettli M. S.; Ryti N. R. I.; Saldiva P. H. N.; Scovronick N.; Seposo X.; Sera F.; Silva S. P.; Stafoggia M.; Tobias A.; Garshick E.; Bernstein A. S.; Zanobetti A.; Schwartz J.; Gasparrini A.; Koutrakis P. Associations Between Extreme Temperatures and Cardiovascular Cause-Specific Mortality: Results From 27 Countries. Circulation 2023, 147 (1), 35–46. 10.1161/CIRCULATIONAHA.122.061832. 36503273 PMC9794133

[ref19] Kim K. N.; Lim Y. H.; Bae S.; Kim J. H.; Hwang S. S.; Kim M. J.; Oh J.; Lim H.; Choi J.; Kwon H. J. Associations between cold spells and hospital admission and mortality due to diabetes: A nationwide multi-region time-series study in Korea. Sci. Total Environ. 2022, 838, 156464 10.1016/j.scitotenv.2022.156464. 35660607

[ref20] Liu J.; Varghese B. M.; Hansen A.; Zhang Y.; Driscoll T.; Morgan G.; Dear K.; Gourley M.; Capon A.; Bi P. Heat exposure and cardiovascular health outcomes: a systematic review and meta-analysis. Lancet Planet Health 2022, 6 (6), e484–e495. 10.1016/S2542-5196(22)00117-6. 35709806

[ref21] Liu P.; Chen Z.; Han S.; Xia X.; Wang L.; Li X. The added effects of cold spells on stroke admissions: Differential effects on ischemic and hemorrhagic stroke. Int. J. Stroke 2024, 19 (2), 217–225. 10.1177/17474930231203129. 37697456

[ref22] Ebi K. L.; Vanos J.; Baldwin J. W.; Bell J. E.; Hondula D. M.; Errett N. A.; Hayes K.; Reid C. E.; Saha S.; Spector J.; Berry P. Extreme Weather and Climate Change: Population Health and Health System Implications. Annu. Rev. Public Health 2021, 42, 293–315. 10.1146/annurev-publhealth-012420-105026. 33406378 PMC9013542

[ref23] Gordon C. J.; Johnstone A. F.; Aydin C. Thermal stress and toxicity. Compr Physiol 2014, 4 (3), 995–1016. 10.1002/cphy.c130046. 24944028

[ref24] Xu R.; Huang S.; Shi C.; Wang R.; Liu T.; Li Y.; Zheng Y.; Lv Z.; Wei J.; Sun H.; Liu Y. Extreme Temperature Events, Fine Particulate Matter, and Myocardial Infarction Mortality. Circulation 2023, 148 (4), 312–323. 10.1161/CIRCULATIONAHA.122.063504. 37486993

[ref25] Huang Y.; Wang Y.; Zhang T.; Wang P.; Huang L.; Guo Y. Exploring Health Effects under Specific Causes of Mortality Based on 90 Definitions of PM_2.5_ and Cold Spell Combined Exposure in Shanghai, China. Environ. Sci. Technol. 2023, 57 (6), 2423–2434. 10.1021/acs.est.2c06461. 36724352

[ref26] Zhao Y.; Hu Y.; Smith J. P.; Strauss J.; Yang G. Cohort profile: the China Health and Retirement Longitudinal Study (CHARLS). Int. J. Epidemiol 2014, 43 (1), 61–8. 10.1093/ije/dys203. 23243115 PMC3937970

[ref27] Guo Q.; Bai X.; Feng N. Social participation and depressive symptoms among Chinese older adults: A study on rural-urban differences. J. Affect Disord 2018, 239, 124–130. 10.1016/j.jad.2018.06.036. 30005325

[ref28] Xie Y.; Hu J. An Introduction to the China Family Panel Studies (CFPS). Chinese Sociological Review 2014, 47 (1), 3–29.

[ref29] Zeng Y.; Feng Q.; Hesketh T.; Christensen K.; Vaupel J. W. Survival, disabilities in activities of daily living, and physical and cognitive functioning among the oldest-old in China: a cohort study. Lancet 2017, 389 (10079), 1619–1629. 10.1016/S0140-6736(17)30548-2. 28285816 PMC5406246

[ref30] Guo Y.; Gasparrini A.; Armstrong B. G.; Tawatsupa B.; Tobias A.; Lavigne E.; Coelho M.; Pan X.; Kim H.; Hashizume M.; Honda Y.; Guo Y. L.; Wu C. F.; Zanobetti A.; Schwartz J. D.; Bell M. L.; Scortichini M.; Michelozzi P.; Punnasiri K.; Li S.; Tian L.; Garcia S. D. O.; Seposo X.; Overcenco A.; Zeka A.; Goodman P.; Dang T. N.; Dung D. V.; Mayvaneh F.; Saldiva P. H. N.; Williams G.; Tong S. Heat Wave and Mortality: A Multicountry, Multicommunity Study. Environ. Health Perspect 2017, 125 (8), 087006 10.1289/EHP1026. 28886602 PMC5783630

[ref31] Yu G.; Yang L.; Liu M.; Wang C.; Shen X.; Fan L.; Zhang J. Extreme Temperature Exposure and Risks of Preterm Birth Subtypes Based on a Nationwide Survey in China. Environ. Health Perspect 2023, 131 (8), 87009 10.1289/EHP10831. 37585350 PMC10431497

[ref32] Fischer E. M.; Schär C. Consistent geographical patterns of changes in high-impact European heatwaves. Nature Geoscience 2010, 3 (6), 398–403. 10.1038/ngeo866.

[ref33] Guo J.; Ruan Y.; Wang Y.; Wang H.; Ma S.; Wan X.; Zhou X.; Tang Z.; He Y.; Zou Z.; Li J. Maternal Exposure to Extreme Cold Events and Risk of Congenital Heart Defects: A Large Multicenter Study in China. Environ. Sci. Technol. 2024, 58 (8), 3737–3746. 10.1021/acs.est.3c10306. 38359432

[ref34] Geng G.; Xiao Q.; Liu S.; Liu X.; Cheng J.; Zheng Y.; Xue T.; Tong D.; Zheng B.; Peng Y.; Huang X.; He K.; Zhang Q. Tracking Air Pollution in China: Near Real-Time PM_2.5_ Retrievals from Multisource Data Fusion. Environ. Sci. Technol. 2021, 55 (17), 12106–12115. 10.1021/acs.est.1c01863. 34407614

[ref35] Liu S.; Geng G.; Xiao Q.; Zheng Y.; Liu X.; Cheng J.; Zhang Q. Tracking Daily Concentrations of PM_2.5_ Chemical Composition in China since 2000. Environ. Sci. Technol. 2022, 56 (22), 16517–16527. 10.1021/acs.est.2c06510. 36318737 PMC9670839

[ref36] Chen W.; Wang X.; Chen J.; You C.; Ma L.; Zhang W.; Li D. Household air pollution, adherence to a healthy lifestyle, and risk of cardiometabolic multimorbidity: Results from the China health and retirement longitudinal study. Sci. Total Environ. 2023, 855, 158896 10.1016/j.scitotenv.2022.158896. 36150596

[ref37] Zhang Y.; Dong T.; Hu W.; Wang X.; Xu B.; Lin Z.; Hofer T.; Stefanoff P.; Chen Y.; Wang X.; Xia Y. Association between exposure to a mixture of phenols, pesticides, and phthalates and obesity: Comparison of three statistical models. Environ. Int. 2019, 123, 325–336. 10.1016/j.envint.2018.11.076. 30557812

[ref38] Carrico C.; Gennings C.; Wheeler D. C.; Factor-Litvak P. Characterization of Weighted Quantile Sum Regression for Highly Correlated Data in a Risk Analysis Setting. J. Agric Biol. Environ. Stat 2015, 20 (1), 100–120. 10.1007/s13253-014-0180-3. 30505142 PMC6261506

[ref39] Knol M. J.; VanderWeele T. J. Recommendations for presenting analyses of effect modification and interaction. Int. J. Epidemiol 2012, 41 (2), 514–20. 10.1093/ije/dyr218. 22253321 PMC3324457

[ref40] Liu L.; Zhang Y.; Yang Z.; Luo S.; Zhang Y. Long-term exposure to fine particulate constituents and cardiovascular diseases in Chinese adults. J. Hazard Mater. 2021, 416, 126051 10.1016/j.jhazmat.2021.126051. 34492892

[ref41] Wang M.; He Y.; Zhao Y.; Zhang L.; Liu J.; Zheng S.; Bai Y. Exposure to PM_2.5_ and its five constituents is associated with the incidence of type 2 diabetes mellitus: a prospective cohort study in northwest China. Environ. Geochem Health 2024, 46 (2), 34 10.1007/s10653-023-01794-3. 38227152

[ref42] Li J.; Tang W.; Li S.; He C.; Dai Y.; Feng S.; Zeng C.; Yang T.; Meng Q.; Meng J.; Pan Y.; Deji S.; Zhang J.; Xie L.; Guo B.; Lin H.; Zhao X. Ambient PM_2.5_ and its components associated with 10-year atherosclerotic cardiovascular disease risk in Chinese adults. Ecotoxicol Environ. Saf 2023, 263, 115371 10.1016/j.ecoenv.2023.115371. 37643506

[ref43] Kirrane E. F.; Luben T. J.; Benson A.; Owens E. O.; Sacks J. D.; Dutton S. J.; Madden M.; Nichols J. L. A systematic review of cardiovascular responses associated with ambient black carbon and fine particulate matter. Environ. Int. 2019, 127, 305–316. 10.1016/j.envint.2019.02.027. 30953813 PMC8517909

[ref44] Ogbomo A. S.; Gronlund C. J.; O’Neill M. S.; Konen T.; Cameron L.; Wahl R. Vulnerability to extreme-heat-associated hospitalization in three counties in Michigan, USA, 2000–2009. Int. J. Biometeorol 2017, 61 (5), 833–843. 10.1007/s00484-016-1261-5. 27796569 PMC5410403

[ref45] Chen J.; Yang J.; Zhou M.; Yin P.; Wang B.; Liu J.; Chen Z.; Song X.; Ou C. Q.; Liu Q. Cold spell and mortality in 31 Chinese capital cities: Definitions, vulnerability and implications. Environ. Int. 2019, 128, 271–278. 10.1016/j.envint.2019.04.049. 31071590

[ref46] Yang J.; Yin P.; Sun J.; Wang B.; Zhou M.; Li M.; Tong S.; Meng B.; Guo Y.; Liu Q. Heatwave and mortality in 31 major Chinese cities: Definition, vulnerability and implications. Sci. Total Environ. 2019, 649, 695–702. 10.1016/j.scitotenv.2018.08.332. 30176480

[ref47] Pan R.; Okada A.; Yamana H.; Yasunaga H.; Kumazawa R.; Matsui H.; Fushimi K.; Honda Y.; Kim Y. Association between ambient temperature and cause-specific cardiovascular disease admissions in Japan: A nationwide study. Environ. Res. 2023, 225, 115610 10.1016/j.envres.2023.115610. 36871945

[ref48] Zhang H.; Liu L.; Zeng Y.; Liu M.; Bi J.; Ji J. S. Effect of heatwaves and greenness on mortality among Chinese older adults. Environ. Pollut. 2021, 290, 118009 10.1016/j.envpol.2021.118009. 34523521

[ref49] Haberzettl P.; O’Toole T. E.; Bhatnagar A.; Conklin D. J. Exposure to Fine Particulate Air Pollution Causes Vascular Insulin Resistance by Inducing Pulmonary Oxidative Stress. Environ. Health Perspect 2016, 124 (12), 1830–1839. 10.1289/EHP212. 27128347 PMC5132639

[ref50] Fouladi F.; Bailey M. J.; Patterson W. B.; Sioda M.; Blakley I. C.; Fodor A. A.; Jones R. B.; Chen Z.; Kim J. S.; Lurmann F.; Martino C.; Knight R.; Gilliland F. D.; Alderete T. L. Air pollution exposure is associated with the gut microbiome as revealed by shotgun metagenomic sequencing. Environ. Int. 2020, 138, 105604 10.1016/j.envint.2020.105604. 32135388 PMC7181344

[ref51] Fiordelisi A.; Piscitelli P.; Trimarco B.; Coscioni E.; Iaccarino G.; Sorriento D. The mechanisms of air pollution and particulate matter in cardiovascular diseases. Heart Fail Rev. 2017, 22 (3), 337–347. 10.1007/s10741-017-9606-7. 28303426

[ref52] Frikke-Schmidt H.; Roursgaard M.; Lykkesfeldt J.; Loft S.; Nøjgaard J. K.; Møller P. Effect of vitamin C and iron chelation on diesel exhaust particle and carbon black induced oxidative damage and cell adhesion molecule expression in human endothelial cells. Toxicol. Lett. 2011, 203 (3), 181–9. 10.1016/j.toxlet.2011.03.011. 21421028

[ref53] Lei X.; Chen R.; Wang C.; Shi J.; Zhao Z.; Li W.; Yan B.; Chillrud S.; Cai J.; Kan H. Personal Fine Particulate Matter Constituents, Increased Systemic Inflammation, and the Role of DNA Hypomethylation. Environ. Sci. Technol. 2019, 53 (16), 9837–9844. 10.1021/acs.est.9b02305. 31328512 PMC7092684

[ref54] Yardley J. E.; Stapleton J. M.; Sigal R. J.; Kenny G. P. Do heat events pose a greater health risk for individuals with type 2 diabetes?. Diabetes Technol. Ther 2013, 15 (6), 520–9. 10.1089/dia.2012.0324. 23530578

[ref55] Wang L.; Liu T.; Hu M.; Zeng W.; Zhang Y.; Rutherford S.; Lin H.; Xiao J.; Yin P.; Liu J.; Chu C.; Tong S.; Ma W.; Zhou M. The impact of cold spells on mortality and effect modification by cold spell characteristics. Sci. Rep 2016, 6, 38380 10.1038/srep38380. 27922084 PMC5138587

[ref56] Chen Z.; Liu P.; Xia X.; Wang L.; Li X. The underlying mechanisms of cold exposure-induced ischemic stroke. Sci. Total Environ. 2022, 834, 155514 10.1016/j.scitotenv.2022.155514. 35472344

[ref57] Schifano P.; Lallo A.; Asta F.; De Sario M.; Davoli M.; Michelozzi P. Effect of ambient temperature and air pollutants on the risk of preterm birth, Rome 2001–2010. Environ. Int. 2013, 61, 77–87. 10.1016/j.envint.2013.09.005. 24103349

[ref58] Rai M.; Stafoggia M.; de’Donato F.; Scortichini M.; Zafeiratou S.; Vazquez Fernandez L.; Zhang S.; Katsouyanni K.; Samoli E.; Rao S.; Lavigne E.; Guo Y.; Kan H.; Osorio S.; Kyselý J.; Urban A.; Orru H.; Maasikmets M.; Jaakkola J. J. K.; Ryti N.; Pascal M.; Hashizume M.; Fook Sheng Ng C.; Alahmad B.; Hurtado Diaz M.; De la Cruz Valencia C.; Nunes B.; Madureira J.; Scovronick N.; Garland R. M.; Kim H.; Lee W.; Tobias A.; Íñiguez C.; Forsberg B.; Åström C.; Maria Vicedo-Cabrera A.; Ragettli M. S.; Leon Guo Y. L.; Pan S. C.; Li S.; Gasparrini A.; Sera F.; Masselot P.; Schwartz J.; Zanobetti A.; Bell M. L.; Schneider A.; Breitner S. Heat-related cardiorespiratory mortality: Effect modification by air pollution across 482 cities from 24 countries. Environ. Int. 2023, 174, 107825 10.1016/j.envint.2023.107825. 36934570

[ref59] Zhou W.; Wang Q.; Li R.; Kadier A.; Wang W.; Zhou F.; Ling L. Combined effects of heatwaves and air pollution, green space and blue space on the incidence of hypertension: A national cohort study. Sci. Total Environ. 2023, 867, 161560 10.1016/j.scitotenv.2023.161560. 36640878

[ref60] Kelly F. J.; Fussell J. C. Linking ambient particulate matter pollution effects with oxidative biology and immune responses. Ann. N.Y. Acad. Sci. 2015, 1340, 84–94. 10.1111/nyas.12720. 25716617

[ref61] Iba T.; Helms J.; Levi M.; Levy J. H. Inflammation, coagulation, and cellular injury in heat-induced shock. Inflamm Res. 2023, 72 (3), 463–473. 10.1007/s00011-022-01687-8. 36609608

[ref62] Soulsby L. K.; Bennett K. M. Marriage and psychological wellbeing: the role of social support. Psychology 2015, 06, 1349–1359. 10.4236/psych.2015.611132.

[ref63] Jennings E. A.; Mkhwanazi N.; Berkman L. Receipt of Emotional Support among Rural South African Adults. Ageing Soc. 2020, 40 (5), 1039–1063. 10.1017/S0144686X18001526. 33223581 PMC7678778

[ref64] Jee Y.; Cho Y. Health behaviors and health status of Korean middleaged men by marital status: Korea Community Health Study, 2015. Epidemiol Health 2019, 41, e2019019 10.4178/epih.e2019019. 31096748 PMC6759495

[ref65] Liu C.; Chan K. H.; Lv J.; Lam H.; Newell K.; Meng X.; Liu Y.; Chen R.; Kartsonaki C.; Wright N.; Du H.; Yang L.; Chen Y.; Guo Y.; Pei P.; Yu C.; Shen H.; Wu T.; Kan H.; Chen Z.; Li L. Long-Term Exposure to Ambient Fine Particulate Matter and Incidence of Major Cardiovascular Diseases: A Prospective Study of 0.5 Million Adults in China. Environ. Sci. Technol. 2022, 56 (18), 13200–13211. 10.1021/acs.est.2c03084. 36044001 PMC9494741

[ref66] Macintyre H. L.; Heaviside C.; Cai X.; Phalkey R. The winter urban heat island: Impacts on cold-related mortality in a highly urbanized European region for present and future climate. Environ. Int. 2021, 154, 106530 10.1016/j.envint.2021.106530. 33895439 PMC8543073

[ref67] Hu Y.; Yao M.; Liu Y.; Zhao B. Personal exposure to ambient PM_2.5_, PM_10_, O_3_, NO_2_, and SO_2_ for different populations in 31 Chinese provinces. Environ. Int. 2020, 144, 106018 10.1016/j.envint.2020.106018. 32771828

[ref68] Zhou B.; Zhao B.; Guo X. F.; Chen R. J.; Kan H. D. Investigating the geographical heterogeneity in PM_10_-mortality associations in the China Air Pollution and Health Effects Study (CAPES): A potential role of indoor exposure to PM_10_ of outdoor origin. Atmos. Environ. 2013, 75, 217–223. 10.1016/j.atmosenv.2013.04.044.

[ref69] Sheppard L.; Burnett R. T.; Szpiro A. A.; Kim S. Y.; Jerrett M.; Pope C. A. 3rd; Brunekreef B. Confounding and exposure measurement error in air pollution epidemiology. Air Qual Atmos Health 2012, 5 (2), 203–216. 10.1007/s11869-011-0140-9. 22662023 PMC3353104

