# Supplementary material for: Interaction between Extreme Temperature Events and Fine Particulate Matter on Cardiometabolic Multimorbidity: Evidence from Four National Cohort Studies
Source: Environ Sci Technol. 2024 Jul 3;58(28):12379–89. doi: 10.1021/acs.est.4c02080 (PMC11256764; doi:10.1021/acs.est.4c02080)
Supplement: Supplementary file 1 — es4c02080_si_001.docx [file es4c02080_si_001.docx]

**Supplementary Information**

Interaction between extreme temperature events and fine particulate matter on cardiometabolic multi-morbidity: evidence from four national cohort studies

Shouxin Peng,^1,2,†^ Zhaoyuan Li,^1,2,†^ John S. Ji,^3^ Bingbing Chen,^1^ Xiaoyi Yin,^1^ Wei Zhang,^1^ Feifei Liu,^1,2^ Huanfeng Shen,^4,*^ Hao Xiang^1,2,*^

**Affiliations**

^1^ Global Health Department, School of Public Health, Wuhan University, Wuhan 430071, China

^2^ Global Health Institute, Wuhan University, Wuhan 430071, China

^3^ Vanke School of Public Health, Tsinghua University, Beijing 100084, China

^4^ School of Resource and Environmental Sciences, Wuhan University, Wuhan 430079, China

^†^ S. P. and Z. L. contributed equally to this work.

**^*^ Corresponding Authors**

**Hao Xiang** - Department of Global Health, School of Public Health, Wuhan University, Wuhan 430071, China; Global Health Institute, Wuhan University, Wuhan 430071, China; Email: xianghao@whu.edu.cn

**Huanfeng Shen** - School of Resource and Environmental Sciences, Wuhan University, Wuhan 430079, China; Email: shenhf@whu.edu.cn

**Numbers of Pages: 22**

**Numbers of Figures: 5**

**Numbers of Tables: 10**

**Supplementary Information Contents**

**Supplement eMethods**

**Table S1**. Definitions of extreme temperature events and annual occurred-events days.

**Table S2**. Baseline characteristics of participants from the four cohorts.

**Table S3**. Spearman correlation matrix between the average levels of annual mean PM_2.5_ and its 5 major constituents' concentrations during the follow-up periods.

**Table S4**. Stratification analysis for the associations between PM_2.5_ constituents and CMM.

**Table S5**. Akaike information criterion values for the association between multi-definitions ETEs and CMM occurrence.

**Table S6**. Associations of exposure to extreme temperature events, PM_2.5_ and its constituents with CMM occurrence under two-year exposure window.

**Table S7**. Associations of exposure to extreme temperature events, PM_2.5_ and its constituents with CMM occurrence after excluding the participants with a follow-up under 3 years.

**Table S8**. Associations of exposure to extreme temperature events, PM_2.5_ and its constituents with CMM occurrence after excluding the participants developed-CMM within 2 years.

**Table S9**. Associations of exposure to extreme temperature events, PM_2.5_ and its constituents with CMM occurrence after adjusting extra covariates.

**Table S10**. Associations of exposure to extreme temperature events, PM_2.5_ and its constituents with CMM occurrence based on a nested case-control study.

**Figure S1**. Flowchart of the current study.

**Figure S2**. The geographic distribution of monitoring stations of NCEI in the current temperature assessment.

**Figure S3**. Directed Acyclic Graph for the association of exposure to extreme temperature events, PM_2.5_ and its constituents with CMM occurrence.

**Figure S4**. Age distribution of current study participants.

**Figure S5**. The weights of 5-kinds PM_2.5_ constituents mixture based on weighted quantile sum regression in positive direction with CMM occurrence.

**Supplement eMethods**

**1. Study design and participants**

Chinese Longitudinal Healthy Longevity Survey (CLHLS) is a national investigation conducted in a randomly selected half of the counties and cities in 23 of the 31 provinces, autonomous regions, and municipalities. It is estimated the survey covers about 85 percent of the total population of China. CLHLS adopted a targeted random-sample design and the old aged 65+ were selected and interviewed face-to-face. Starting its first investigation in 1998, this cohort was followed up in 2000, 2002, 2005, 2008, 2011/2012, 2014, and 2018. In this study, we selected the fifth wave (2008), sixth (2011/2012), and seventh (2014) surveys for analysis. More details about the sample design and interview can be found elsewhere.^1^ The database is available at the Peking University Open Research Data Platform (https://opendata.pku.edu.cn/dataverse/CHADS).

China Family Panel Studies (CFPS) is a nationally representative survey in China. It adopted a stratified multi-stage probability strategy covering 25 provinces, autonomous regions, and municipalities. Participants were recruited in 2010 (the baseline year) and followed up in 2012, 2014, 2016, and 2018. Information about participants was collected through questionnaires in each survey. A detailed introduction to CFPS can be found elsewhere.^2^ The database is available at the Peking University Open Research Data Platform (https://opendata.pku.edu.cn/dataverse/CFPS/).

China Health and Retirement Longitudinal Study (CHARLS) adopted a multistage probability sampling strategy covering 150 counties/districts across 28 provinces, autonomous regions, and municipalities in China. Participants aged 45+ were recruited and interviewed in 2011 as the first wave. The second, third, and fourth waves were in 2013, 2015, and 2018, respectively. More information can be found in the published cohort profile.^3^ The database is available at the Peking University Open Research Data Platform (https://opendata.pku.edu.cn/dataverse/CHARLS/).

China Longitudinal Aging Social Survey (CLASS) adopted a multistage probability sampling method covering 134 counties/districts across 28 provinces, autonomous regions, and municipalities in China. The nationwide survey was first conducted in 2014 as the baseline. Two follow-ups were conducted in 2016 and 2018. The subjects are the old population aged 60 years old and above. More details about the sample design and interview can be found elsewhere.^4^ The database is available at the National Survey Research Center at Renmin University of China (http://class.ruc.edu.cn/).

**2. Exposure measurement**

Monthly gridded ozone concentrations with a 10 km spatial resolution were derived from ChinaHighO_3_ dataset.^5^ Specifically, ambient ozone concentrations were generated from OMI Total-column O_3_ products together with other auxiliary data (e.g., ground-based measurements, satellite remote sensing products, atmospheric reanalysis, and model simulations) using artificial intelligence by considering the spatiotemporal heterogeneity of air pollution. The data we obtained was the OMI Level 3 (L3) yearly 0.25-degree (≈25 km) gridded ground-level O_3_ concentration measurements in China from 2010 to 2020, averaged from the Level 2 daily (local time 13:30) products. This dataset had high accuracy with a cross-validation coefficient of determination (CV-R^2^) of 0.87 and a root-mean-square error (RMSE) of 17.10 μg/m^3^ in China.

**3. Definitions of outcomes**

In CLHLS, CHARLS, and CLASS, the question is "Are you suffering from any of the following diseases?" Participants are required to answer yes or no to each disease. There could be some slight differences in the category of each disease and how they were asked, but the participants can totally understand and answer correctly. The names of targeted diseases in this study are heart disease, diabetes or high blood sugar, and stroke. In CFPS, the question is "List the name of two major chronic diseases you were diagnosed as". According to the Disease Classification Codebook for CFPS, diabetes is encoding as 5.37, stroke is encoding as 11.67. Heat disease includes a large category (11.6-11.65). Hypertension is not often listed in the answer to this question if the participant has other major diseases as well. Therefore, we did not report the hypertension prevalence in Table 1.

**4. Residence information**

In CLHLS, personal residential address information was unavailable because of privacy. To deal with this situation, we adopted a widely used method that has been published elsewhere.^6, 7^ The 2010 population size (total, male and female) of the city was described in the community questionnaire in the 2008, 2011/2012, and 2014 surveys. With reference to *Tabulation on the 2010 Population Census of China by County*, we identified the city name where each participant belonged.

In CFPS, CHARLS, and CLASS, city-level address information of each participant was provided in the datasets.

**5. Covariates selection**

We developed a directed acyclic graph (DAG) to identify potential covariates that need to be adjusted in our multivariate regression analyses, using an online DAGitty tool (https://www.dagitty.net/). Based on existing literatures,^8-11^ we included a rich set of covariates in the DAG that should be considered in the current analyses (Figure. S3). A minimally sufficient adjustment set, involving age, gender, marital status, education, smoking status, residence type and region, was firstly selected. Besides, the body mass index (BMI) was also selected based the previous studies.^8-11^ Finally, we included the above eight covariates into the fully adjusted model. Other covariates, such as drinking status, physical exercise, sleep quality and hypertension, were considered at the sensitivity analysis.

**6. Statistical analysis**

We calculated the Pearson correlation coefficients between the five PM_2.5_ chemical constituents. The weighted quantile sum (WQS) regression model with a logistic conjunction was performed to examine the joint association of five highly correlated components with CMM.^12, 13^ WQS regression model generated a weighted linear index (WQS index) along with the weight score for each individual component, which represented certain pollutants contribution (ranging from 0 to 1 and sum to 1) to the WQS index. The whole data was randomly split into training and validation sets with proportion of 4:6 in the WQS analysis. We then estimated the weights with a bootstrap method with 10 000 items focusing on the positive direction to avoid the reversal paradox.^14^ Covariates in the WQS analysis were selected and adjusted based on the aforementioned DAG method.

The formulas of relative excess risk due to interaction (RERI), attributable proportion due to interaction (AP) and the synergy index (S) for additive interaction effect are as follows:

$$RERI ={HR}_{11}-{HR}_{10}-{HR}_{01}+1$$

HR_11_ represents the HR value in the X_11_ scenario, HR_10_ represents the HR value in the X_10_ scenario. HR_01_ represents the HR value in the X_01_ scenario. RERI = 0 indicates that there is no interaction between two variables, and the combined exposure effect is directly equal to the sum of their exposure effects. RERI > 0 indicates that there is synergistic effect. RERI < 0 indicates that there is antagonistic effect.

$$AP=\frac{RERI}{{HR}_{11}}$$

Proportion attributable (AP) refers to the proportion of the combined effect caused by the interaction. AP = 0 indicates no interaction. AP > 0 indicates there is synergistic effect. AP < 0 indicates that there is antagonistic effect. The value of AP ranges from -1 to 1.

$$S= \frac{{HR}_{11}-1}{{(HR}_{10}-1)+({HR}_{01}-1)}$$

Synergy index (S) is the ratio between the combined effect and the individual effect index, S = 1 indicates there is no interaction or; S >1 indicates there is synergistic effect. S < 1 indicates there is antagonistic effect. The values of S range from 0 to positive infinity.

**References**

1. Zeng, Y.; Feng, Q.; Hesketh, T.; Christensen, K.; Vaupel, J. W., Survival, disabilities in activities of daily living, and physical and cognitive functioning among the oldest-old in China: a cohort study. *Lancet* **2017,** *389*, (10079), 1619-1629.

2. Xie, Y.; Hu, J., An Introduction to the China Family Panel Studies (CFPS). *Chinese Sociological Review* **2014,** *47*, (1), 3-29.

3. Zhao, Y.; Hu, Y.; Smith, J. P.; Strauss, J.; Yang, G., Cohort profile: the China Health and Retirement Longitudinal Study (CHARLS). *Int J Epidemiol* **2014,** *43*, (1), 61-8.

4. Guo, Q.; Bai, X.; Feng, N., Social participation and depressive symptoms among Chinese older adults: A study on rural-urban differences. *J Affect Disord* **2018,** *239*, 124-130.

5. Wei, J.; Li, Z.; Li, K.; Dickerson, R. R.; Pinker, R. T.; Wang, J.; Liu, X.; Sun, L.; Xue, W.; Cribb, M., Full-coverage mapping and spatiotemporal variations of ground-level ozone (O_3_) pollution from 2013 to 2020 across China. *Remote Sensing of Environment* **2022,** *270*.

6. Hu, K.; Keenan, K.; Hale, J. M.; Borger, T., The association between city-level air pollution and frailty among the elderly population in China. *Health Place* **2020,** *64*, 102362.

7. Zhou, W.; Wang, Q.; Li, R.; Kadier, A.; Wang, W.; Zhou, F.; Ling, L., Combined effects of heatwaves and air pollution, green space and blue space on the incidence of hypertension: A national cohort study. *Sci Total Environ* **2023,** *867*, 161560.

8. Luo, H.; Zhang, Q.; Yu, K.; Meng, X.; Kan, H.; Chen, R., Long-term exposure to ambient air pollution is a risk factor for trajectory of cardiometabolic multimorbidity: A prospective study in the UK Biobank. *EBioMedicine* **2022,** *84*, 104282.

9. Su, B.; Liu, C.; Chen, L.; Wu, Y.; Li, J.; Zheng, X., Long-term exposure to PM_2.5_ and O_3_ with cardiometabolic multimorbidity: Evidence among Chinese elderly population from 462 cities. *Ecotoxicol Environ Saf* **2023,** *255*, 114790.

10. Zou, H.; Zhang, S.; Cai, M.; Qian, Z. M.; Zhang, Z.; Chen, L.; Wang, X.; Arnold, L. D.; Howard, S. W.; Li, H.; Lin, H., Ambient air pollution associated with incidence and progression trajectory of cardiometabolic diseases: A multi-state analysis of a prospective cohort. *Sci Total Environ* **2023,** *862*, 160803.

11. Jiang, Z.; Zhang, S.; Chen, K.; Wu, Y.; Zeng, P.; Wang, T., Long-term influence of air pollutants on morbidity and all-cause mortality of cardiometabolic multi-morbidity: A cohort analysis of the UK Biobank participants. *Environ Res* **2023,** *237*, (Pt 1), 116873.

12. Zhang, Y.; Dong, T.; Hu, W.; Wang, X.; Xu, B.; Lin, Z.; Hofer, T.; Stefanoff, P.; Chen, Y.; Wang, X.; Xia, Y., Association between exposure to a mixture of phenols, pesticides, and phthalates and obesity: Comparison of three statistical models. *Environ Int* **2019,** *123*, 325-336.

13. Carrico, C.; Gennings, C.; Wheeler, D. C.; Factor-Litvak, P., Characterization of Weighted Quantile Sum Regression for Highly Correlated Data in a Risk Analysis Setting. *J Agric Biol Environ Stat* **2015,** *20*, (1), 100-120.

14. Li, W.; Xiao, H.; Wu, H.; Pan, C.; Deng, K.; Xu, X.; Zhang, Y., Analysis of environmental chemical mixtures and nonalcoholic fatty liver disease: NHANES 1999-2014. *Environ Pollut* **2022,** *311*, 119915.

**Table S1**. Definitions of extreme temperature events and annual occurred-events days.

| Name | Definitions | |  | Annual occurred-events days | | | | |
| --- | --- | --- | --- | --- | --- | --- | --- | --- |
|  | Threshold | Duration (days) |  | Min | Median | Max | Mean ± SD |  |
| HW01 | 90^th^ | 2 |  | 9 | 38 | 67 | 37.03 ± 11.85 |  |
| HW02 | 90^th^ | 3 |  | 4 | 34 | 65 | 32.76 ± 12.17 |  |
| HW03 | 90^th^ | 4 |  | 0 | 30 | 61 | 28.51 ± 12.11 |  |
| HW04 | 92.5^th^ | 2 |  | 4 | 28 | 56 | 27.04 ± 10.89 |  |
| HW05 | 92.5^th^ | 3 |  | 0 | 23 | 55 | 23.15 ± 10.76 |  |
| HW06 | 92.5^th^ | 4 |  | 0 | 20 | 53 | 19.63 ± 10.64 |  |
| HW07 | 95^th^ | 2 |  | 0 | 16 | 46 | 17.08 ± 8.99 |  |
| HW08 | 95^th^ | 3 |  | 0 | 13 | 46 | 13.91 ± 8.94 |  |
| HW09 | 95^th^ | 4 |  | 0 | 10 | 43 | 11.34 ± 8.73 |  |
| HW10 | 97.5^th^ | 2 |  | 0 | 6 | 31 | 7.34 ± 6.37 |  |
| HW11 | 97.5^th^ | 3 |  | 0 | 4 | 31 | 5.55 ± 5.99 |  |
| HW12 | 97.5^th^ | 4 |  | 0 | 1 | 26 | 4.25 ± 5.47 |  |
| CS01 | 10^th^ | 2 |  | 6 | 31 | 71 | 29.03 ± 11.44 |  |
| CS02 | 10^th^ | 3 |  | 0 | 28 | 68 | 25.72 ± 12.10 |  |
| CS03 | 10^th^ | 4 |  | 0 | 26 | 67 | 22.89 ± 12.32 |  |
| CS04 | 7.5^th^ | 2 |  | 0 | 24 | 63 | 21.27 ± 11.04 |  |
| CS05 | 7.5^th^ | 3 |  | 0 | 22 | 61 | 18.87 ± 11.26 |  |
| CS06 | 7.5^th^ | 4 |  | 0 | 19 | 55 | 16.53 ± 10.93 |  |
| CS07 | 5^th^ | 2 |  | 0 | 16 | 43 | 14.43 ± 9.45 |  |
| CS08 | 5^th^ | 3 |  | 0 | 14 | 42 | 12.41±9.26 |  |
| CS09 | 5^th^ | 4 |  | 0 | 12 | 39 | 10.70 ± 8.85 |  |
| CS10 | 2.5^th^ | 2 |  | 0 | 8 | 28 | 7.36 ± 6.29 |  |
| CS11 | 2.5^th^ | 3 |  | 0 | 6 | 24 | 6.15 ± 5.78 |  |
| CS12 | 2.5^th^ | 4 |  | 0 | 5 | 21 | 5.23 ± 5.26 |  |

Abbreviations: HW, heat wave; CS, cold spell; Min, minimum; Max, maximum; SD, standard deviation.

**Table S2**. Baseline characteristics of participants from the four cohorts.

| **Characteristics** | | | **CHARLS** | **CLASS** | **CFPS** | **CLHLS** |
| --- | --- | --- | --- | --- | --- | --- |
| **Population** | | |  |  |  |  |
|  | No. of participants | | 11136 | 7368 | 38476 | 7160 |
|  | No. of CMM | | 430 (3.9%) | 195 (2.7%) | 364 (1.0%) | 240 (3.4%) |
|  | Total person-years | | 60343 | 16773 | 290591 | 32536 |
|  | Median follow-up years | | 6.00 | 1.92 | 8.08 | 5.50 |
| **Demographic factors** | | |  |  |  |  |
|  | Age (years) | | 58.06 (9.39) | 69.14 (7.32) | 41.06 (16.86) | 82.72 (11.26) |
|  | Gender (Male) | | 5515 (49.5%) | 3833 (52.0%) | 19246 (50.0%) | 3292 (46.0%) |
|  | BMI (kg/m^2^) | | 23.2 (3.50) | 22.28 (2.49) | 22.09 (3.36) | 20.67 (3.52) |
|  | Marital status (Married) | | 9909 (89.0%) | 5354 (72.7%) | 28673 (74.5%) | 4232 (59.1%) |
|  | Education | |  |  |  |  |
|  |  | Low | 3060 (27.5%) | 2019 (27.4%) | 9528 (24.8%) | 4,151 (58.0%) |
|  |  | Middle | 4555 (40.9%) | 3074 (41.7%) | 7986 (20.8%) | 2,240 (31.3%) |
|  |  | High | 3521 (31.6%) | 2275 (30.9%) | 20962 (54.5%) | 769 (10.7%) |
|  | Urbanity | | 3866 (34.7%) | 3850 (52.3%) | 21654 (56.3%) | 4736 (66.1%) |
|  | Region (North) | | 4722 (42.4%) | 3005 (40.8%) | 20114 (52.3%) | 2595 (36.2%) |
| **Health status** | | |  |  |  |  |
|  | Hypertension ^a^ | | 2173 (19.5%) | 1722 (23.4%) | ̶ | 1278 (17.8%) |
| **Behavioral factors** | | |  |  |  |  |
|  | Smoker (former or current) | | 3702 (33.2%) | 2689 (36.5%) | 11235 (29.2%) | 1528 (21.3%) |
|  | Drinker (dormer or current) ^b^ | | 3973 (35.7%) | ̶ | 5744 (14.9%) | 1508 (21.1%) |
|  | Physical exercise ^c^ | | 5205 (53.7%) | 1104 (15%) | 12360 (32.1%) | 2126 (29.7%) |
|  | Enough sleep ^d^ | | 5381 (50.1%) | ̶ | 29418 (85.8%) | 5329 (74.6%) |
| **Environmental factors** | | |  |  |  |  |
|  | PM_2.5_ (µg/m^3^) | | 41.06 (15.26) | 38.37 (11.21) | 37.63 (15.28) | 58.65 (20.82) |
|  |  | NO_3_^-^ (µg/m^3^) | 9.20 (4.26) | 8.57 (3.39) | 8.28 (4.00) | 12.70 (5.43) |
|  |  | SO_4_^2-^ (µg/m^3^) | 7.57 (2.61) | 6.84 (1.81) | 6.91 (2.73) | 11.28 (3.60) |
|  |  | NH_4_^+^ (µg/m^3^) | 6.22 (2.53) | 5.71 (1.93) | 5.57 (2.42) | 8.93 (3.30) |
|  |  | OM (µg/m^3^) | 10.14 (3.19) | 9.53 (2.29) | 9.11 (3.24) | 14.07 (4.32) |
|  |  | BC (µg/m^3^) | 1.97 (0.57) | 1.82 (0.42) | 1.77 (0.62) | 2.89 (0.77) |
|  | HW11 (days) | | 6.17 (4.69) | 6.22 (5.73) | 4.50 (5.49) | 9.53 (8.27) |
|  | CS10 (days) | | 10.07 (4.22) | 15.18 (2.87) | 5.18 (5.97) | 6.82 (5.10) |

Abbreviations: CHARLS, China Health and Retirement Longitudinal Study; CLASS, China Longitudinal Aging Social Survey; CFPS, China Family Panel Studies; CLHLS, Chinese Longitudinal Healthy Longevity Study; CMM, cardiometabolic multimorbidity; BMI, body mass index. PM_2.5_, fine particulate matter; NO_3_^-^, nitrate; SO_4_^2-^, sulfate; NH_4_^+^, ammonium; OM, organic matter; BC, black carbon; HW11, heat wave frequency for the definition of daily average temperature equal to or higher than 97.5^th^ percentile for at least 3 consecutive days; CS10, cold spell frequency for the definition of daily average temperature lower than 2.5^th^ percentile with at least 2 consecutive days.

Notes:

^a^ 38440 missing data from CFPS.

^b^ 7368 missing data from CLASS.

^c^ 1479 missing data from CHARLS (1439), CFPS (28), and CLHLS (12).

^d^ 11986 missing data from CHARLS (404), CLASS (7368), CFPS (4194), and CLHLS (20).

**Table S3**. Spearman correlation matrix between the average levels of annual mean PM_2.5_ and its 5 major constituents' concentrations during the follow-up periods.

|  | PM_2.5_ | BC | OM | NH_4_^+^ | SO_4_^2-^ | NO_3_^-^ |
| --- | --- | --- | --- | --- | --- | --- |
| PM_2.5_ | 1.000 |  |  |  |  |  |
| BC | 0.938 | 1.000 |  |  |  |  |
| OM | 0.969 | 0.973 | 1.000 |  |  |  |
| NH_4_^+^ | 0.976 | 0.893 | 0.937 | 1.000 |  |  |
| SO_4_^2-^ | 0.974 | 0.956 | 0.958 | 0.968 | 1.000 |  |
| NO_3_^-^ | 0.965 | 0.845 | 0.907 | 0.989 | 0.937 | 1.000 |

Abbreviations: PM_2.5_, fine particulate matter; CMM, cardiometabolic multimorbidity; HR, hazard ratio; CIs, confidence interval; NO_3_^-^, nitrate; SO_4_^2-^, sulfate; NH_4_^+^, ammonium; OM, organic matter; BC, black carbon.

**Table S4**. Stratification analysis for the associations between PM_2.5_ constituents and CMM.

|  |  | NO_3_^-^ | |  | NH_4_^+^ | |  | SO_4_^2-^ | |  | OM | |  | BC | |
| --- | --- | --- | --- | --- | --- | --- | --- | --- | --- | --- | --- | --- | --- | --- | --- |
|  |  | HR (95%CI) | *p*-Z_test_ |  | HR (95%CI) | *p*-Z_test_ |  | HR (95%CI) | *p*-Z_test_ |  | HR (95%CI) | *p*-Z_test_ |  | HR (95%CI) | *p*-Z_test_ |
| Gender | |  |  |  |  |  |  |  |  |  |  |  |  |  |  |
|  | Male | 1.041 (1.019, 1.063) |  |  | 1.078 (1.042, 1.115) |  |  | 1.081 (1.050, 1.113) |  |  | 1.078 (1.053, 1.104) |  |  | 1.048 (1.035, 1.062) |  |
|  | Female | 1.054 (1.035, 1.072) | 0.397 |  | 1.100 (1.069, 1.131) | 0.370 |  | 1.103 (1.076, 1.131) | 0.296 |  | 1.097 (1.076, 1.120) | 0.261 |  | 1.059 (1.048, 1.071) | 0.205 |
| Age, years | |  |  |  |  |  |  |  |  |  |  |  |  |  |  |
|  | <45 | 1.012 (0.921, 1.112) |  |  | 1.048 (0.898, 1.223) |  |  | 1.073 (0.936, 1.230) |  |  | 1.112 (0.997, 1.241) |  |  | 1.075 (1.014, 1.140) |  |
|  | 45~65 | 1.096 (1.071, 1.121) | 0.107 |  | 1.203 (1.158, 1.249) | 0.089 |  | 1.230 (1.189, 1.274) | 0.057 |  | 1.199 (1.167, 1.233) | 0.192 |  | 1.121 (1.105, 1.138) | 0.174 |
|  | >65 | 1.044 (1.026, 1.062) | 0.525 |  | 1.077 (1.048, 1.107) | 0.728 |  | 1.079 (1.055, 1.104) | 0.929 |  | 1.073 (1.053, 1.094) | 0.529 |  | 1.044 (1.034, 1.055) | 0.333 |
| BMI, kg/m^2^ | |  |  |  |  |  |  |  |  |  |  |  |  |  |  |
|  | Underweight | 1.071 (1.022, 1.124) | 0.284 |  | 1.130 (1.047, 1.219) | 0.250 |  | 1.121 (1.049, 1.197) | 0.282 |  | 1.093 (1.033, 1.158) | 0.551 |  | 1.062 (1.031, 1.094) | 0.329 |
|  | Normal | 1.042 (1.023, 1.061) |  |  | 1.077 (1.047, 1.108) |  |  | 1.078 (1.052, 1.105) |  |  | 1.073 (1.052, 1.095) |  |  | 1.045 (1.034, 1.056) |  |
|  | Overweight | 1.054 (1.030, 1.079) | 0.446 |  | 1.105 (1.064, 1.147) | 0.296 |  | 1.115 (1.078, 1.152) | 0.115 |  | 1.113 (1.085, 1.143) | **0.030** |  | 1.068 (1.053, 1.084) | **0.017** |
| Education level | |  |  |  |  |  |  |  |  |  |  |  |  |  |  |
|  | Illiterate | 1.070 (1.047, 1.094) |  |  | 1.136 (1.097, 1.177) |  |  | 1.132 (1.098, 1.168) |  |  | 1.117 (1.089, 1.145) |  |  | 1.074 (1.060, 1.089) |  |
|  | Element | 1.067 (1.041, 1.094) | 0.873 |  | 1.117 (1.074, 1.161) | 0.533 |  | 1.117 (1.080, 1.156) | 0.574 |  | 1.098 (1.069, 1.128) | 0.373 |  | 1.056 (1.041, 1.071) | 0.094 |
|  | Middle or above | 1.046 (1.019, 1.074) | 0.197 |  | 1.091 (1.046, 1.137) | 0.144 |  | 1.100 (1.062, 1.141) | 0.241 |  | 1.094 (1.063, 1.125) | 0.279 |  | 1.056 (1.040, 1.072) | 0.097 |
| Smoke status | |  |  |  |  |  |  |  |  |  |  |  |  |  |  |
|  | Yes | 1.045 (1.016, 1.075) |  |  | 1.092 (1.044, 1.143) |  |  | 1.100 (1.057, 1.144) |  |  | 1.099 (1.064, 1.136) |  |  | 1.060 (1.042, 1.079) |  |
|  | No | 1.048 (1.032, 1.064) | 0.879 |  | 1.088 (1.062, 1.115) | 0.888 |  | 1.090 (1.067, 1.114) | 0.697 |  | 1.086 (1.067, 1.104) | 0.505 |  | 1.052 (1.043, 1.062) | 0.448 |
| Marital status | |  |  |  |  |  |  |  |  |  |  |  |  |  |  |
|  | Married | 1.029 (1.011, 1.048) |  |  | 1.051 (1.022, 1.082) |  |  | 1.054 (1.027, 1.081) |  |  | 1.054 (1.033, 1.076) |  |  | 1.031 (1.020, 1.043) |  |
|  | Others ^a^ | 1.076 (1.054, 1.099) | **0.001** |  | 1.149 (1.113, 1.187) | **<0.001** |  | 1.153 (1.121, 1.185) | **<0.001** |  | 1.136 (1.111, 1.161) | **<0.001** |  | 1.085 (1.072, 1.097) | **<0.001** |
| Residence type | |  |  |  |  |  |  |  |  |  |  |  |  |  |  |
|  | Urban | 1.027 (1.007, 1.047) |  |  | 1.053 (1.021, 1.086) |  |  | 1.064 (1.036, 1.094) |  |  | 1.076 (1.052, 1.100) |  |  | 1.048 (1.035, 1.060) |  |
|  | Rural | 1.073 (1.053, 1.095) | **0.001** |  | 1.135 (1.100, 1.171) | **0.001** |  | 1.128 (1.098, 1.158) | **0.003** |  | 1.105 (1.082, 1.129) | 0.084 |  | 1.062 (1.050, 1.074) | 0.104 |
| Region type | |  |  |  |  |  |  |  |  |  |  |  |  |  |  |
|  | North | 1.027 (1.010, 1.043) |  |  | 1.054 (1.027, 1.081) |  |  | 1.060 (1.036, 1.084) |  |  | 1.070 (1.051, 1.089) |  |  | 1.045 (1.034, 1.055) |  |
|  | South | 1.107 (1.078, 1.135) | **<0.001** |  | 1.190 (1.143, 1.240) | **<0.001** |  | 1.182 (1.141, 1.224) | **<0.001** |  | 1.154 (1.120, 1.190) | **<0.001** |  | 1.077 (1.061, 1.093) | **0.001** |

Abbreviations: PM_2.5_, fine particulate matter; CMM, cardiometabolic multimorbidity; HR, hazard ratio; 95%CI, 95% confidence intervals; NO_3_^-^, nitrate; SO_4_^2-^, sulfate; NH_4_^+^, ammonium; OM, organic matter; BC, black carbon.

Notes: The bold fonts indicate the Z-test *p*-value < 0.05, suggesting that the differences between subgroups are statistically significant. Each stratification controlled for all factors (age, gender, BMI, marital status, education level, smoking status, residence and region type) except the stratification factor itself.

^a^ Others included unmarried, separated, divorced or widowed.

**Table S5.** Akaike information criterion values for the association between multi-definitions ETEs and CMM occurrence.

| Heatwaves | | |  | Cold Spells | | |
| --- | --- | --- | --- | --- | --- | --- |
|  | HR (95%CI) | AIC |  |  | HR (95%CI) | AIC |
| HW01 | 1.006 (1.001, 1.011) | 24150.53 |  | CS01 | 1.073 (1.066, 1.080) | 23872.47 |
| HW02 | 1.007 (1.002, 1.012) | 24148.95 |  | CS02 | 1.064 (1.058, 1.071) | 23704.13 |
| HW03 | 1.009 (1.004, 1.014) | 24143.00 |  | CS03 | 1.063 (1.057, 1.069) | 23711.69 |
| HW04 | 1.007 (1.002, 1.013) | 24149.51 |  | CS04 | 1.073 (1.065, 1.080) | 23695.24 |
| HW05 | 1.007 (1.001, 1.012) | 24150.13 |  | CS05 | 1.066 (1.060, 1.073) | 23735.86 |
| HW06 | 1.008 (1.003, 1.014) | 24146.59 |  | CS06 | 1.065 (1.059, 1.072) | 23756.29 |
| HW07 | 1.009 (1.003, 1.016) | 24148.19 |  | CS07 | 1.076 (1.068, 1.084) | 23752.07 |
| HW08 | 1.012 (1.005, 1.018) | 24143.09 |  | CS08 | 1.074 (1.066, 1.082) | 23774.77 |
| HW09 | 1.013 (1.007, 1.019) | 24140.78 |  | CS09 | 1.064 (1.056, 1.072) | 23873.73 |
| HW10 | 1.016 (1.008, 1.025) | 24142.90 |  | CS10 | 1.091 (1.080, 1.102) | 23655.18 |
| HW11 | 1.019 (1.010, 1.028) | 24138.67 |  | CS11 | 1.080 (1.069, 1.092) | 23958.84 |
| HW12 | 1.015 (1.005, 1.024) | 24147.04 |  | CS12 | 1.085 (1.072, 1.098) | 23986.50 |

Abbreviations: CMM, cardiometabolic multimorbidity; HR, hazard ratio; CI, confidence intervals; ETEs, extreme temperature events involve heatwave and cold spell; AIC, Akaike information criterion.

Notes: The regression model was fully adjusted for age, gender, body mass index, marital status, education, smoking status, residence type, and region. HRs for CMM were estimated by each a ETEs day increment.

**Table S6**. Associations of exposure to extreme temperature events, PM_2.5_ and its constituents with CMM occurrence under two-year exposure window.

|  | Increment | HR (95%CI) | *p*-value |
| --- | --- | --- | --- |
| PM_2.5_ | 10 μg/m^3^ | 1.333 (1.282, 1.385) | < 0.001 |
| NO_3_^-^ | 1 μg/m^3^ | 1.100 (1.083, 1.118) | < 0.001 |
| SO_4_^2-^ | 1 μg/m^3^ | 1.173 (1.149, 1.198) | < 0.001 |
| NH_4_^+^ | 1 μg/m^3^ | 1.181 (1.052, 1.211) | < 0.001 |
| OM | 1 μg/m^3^ | 1.140 (1.122, 1.160) | < 0.001 |
| BC | 1 μg/m^3^ | 1.077 (1.068, 1.087) | < 0.001 |
| HW11 | 1 day | 1.010 (1.004, 1.016) | 0.001 |
| CS10 | 1 day | 1.072 (1.062, 1.082) | <0.001 |

Abbreviations: PM_2.5_, fine particulate matter; CMM, cardiometabolic multimorbidity; HR, hazard ratio; CIs, confidence interval; NO_3_^-^, nitrate; SO_4_^2-^, sulfate; NH_4_^+^, ammonium; OM, organic matter; BC, black carbon; HW11, heat wave frequency for the definition of daily average temperature equal to or higher than 97.5^th^ percentile for at least 3 consecutive days; CS10, cold spell frequency for the definition of daily average temperature lower than 2.5^th^ percentile with at least 2 consecutive days.

Notes: The regression model was fully adjusted for age, gender, body mass index, marital status, education, smoking status, residence type, and region.

**Table S7**. Associations of exposure to extreme temperature events, PM_2.5_ and its constituents with CMM occurrence after excluding the participants with a follow-up under 3 years.

|  | Increment | HR (95%CI) | *p*-value |
| --- | --- | --- | --- |
| PM_2.5_ | 10 μg/m^3^ | 1.398 (1.338, 1.461) | < 0.001 |
| NO_3_^-^ | 1 μg/m^3^ | 1.121 (1.101, 1.142) | < 0.001 |
| SO_4_^2-^ | 1 μg/m^3^ | 1.214 (1.185, 1.245) | < 0.001 |
| NH_4_^+^ | 1 μg/m^3^ | 1.222 (1.188, 1.258) | < 0.001 |
| OM | 1 μg/m^3^ | 1.168 (1.147, 1.191) | < 0.001 |
| BC | 1 μg/m^3^ | 1.096 (1.085, 1.107) | < 0.001 |
| HW11 | 1 day | 1.017 (1.007, 1.027) | 0.001 |
| CS10 | 1 day | 1.081 (1.068, 1.093) | <0.001 |

Abbreviations: PM_2.5_, fine particulate matter; CMM, cardiometabolic multimorbidity; HR, hazard ratio; CIs, confidence interval; NO_3_^-^, nitrate; SO_4_^2-^, sulfate; NH_4_^+^, ammonium; OM, organic matter; BC, black carbon; HW11, heat wave frequency for the definition of daily average temperature equal to or higher than 97.5^th^ percentile for at least 3 consecutive days; CS10, cold spell frequency for the definition of daily average temperature lower than 2.5^th^ percentile with at least 2 consecutive days.

Notes: The regression model was fully adjusted for age, gender, body mass index, marital status, education, smoking status, residence type, and region.

**Table S8**. Associations of exposure to extreme temperature events, PM_2.5_ and its constituents with CMM occurrence after excluding the participants developed-CMM within 2 years.

|  | Increment | HR (95%CI) | *p*-value |
| --- | --- | --- | --- |
| PM_2.5_ | 10 μg/m^3^ | 1.238 (1.192, 1.285) | < 0.001 |
| NO_3_^-^ | 1 μg/m^3^ | 1.059 (1.044, 1.075) | < 0.001 |
| SO_4_^2-^ | 1 μg/m^3^ | 1.126 (1.103, 1.149) | < 0.001 |
| NH_4_^+^ | 1 μg/m^3^ | 1.118 (1.092, 1.144) | < 0.001 |
| OM | 1 μg/m^3^ | 1.114 (1.096, 1.132) | < 0.001 |
| BC | 1 μg/m^3^ | 1.071 (1.062, 1.081) | < 0.001 |
| HW11 | 1 day | 1.009 (1.000, 1.019) | 0.062 |
| CS10 | 1 day | 1.079 (1.068, 1.091) | <0.001 |

Abbreviations: PM_2.5_, fine particulate matter; CMM, cardiometabolic multimorbidity; HR, hazard ratio; CIs, confidence interval; NO_3_^-^, nitrate; SO_4_^2-^, sulfate; NH_4_^+^, ammonium; OM, organic matter; BC, black carbon; HW11, heat wave frequency for the definition of daily average temperature equal to or higher than 97.5^th^ percentile for at least 3 consecutive days; CS10, cold spell frequency for the definition of daily average temperature lower than 2.5^th^ percentile with at least 2 consecutive days.

Notes: The regression model was fully adjusted for age, gender, body mass index, marital status, education, smoking status, residence type, and region.

**Table S9**. Associations of exposure to extreme temperature events, PM_2.5_ and its constituents with CMM occurrence after adjusting extra covariates.

|  | Main model |  | + Drinking status ^a^ |  | + Physical exercise ^b^ |  | + Sleep status ^c^ |  | + Hypertension ^d^ |  | + Ozone pollution |
| --- | --- | --- | --- | --- | --- | --- | --- | --- | --- | --- | --- |
| PM_2.5_ | 1.179 (1.139, 1.220) |  | 1.471 (1.410, 1.535) |  | 1.339 (1.288, 1.392) |  | 1.469 (1.407, 1.533) |  | 1.167 (1.128, 1.209) |  | 1.319 (1.272, 1.369) |
| NO_3_^-^ | 1.047 (1.033, 1.062) |  | 1.144 (1.124, 1.165) |  | 1.106 (1.089, 1.124) |  | 1.143 (1.122, 1.164) |  | 1.043 (1.029, 1.057) |  | 1.115 (1.098, 1.132) |
| SO_4_^2-^ | 1.092 (1.071, 1.113) |  | 1.249 (1.220, 1.279) |  | 1.178 (1.153, 1.204) |  | 1.248 (1.219, 1.278) |  | 1.087 (1.067, 1.108) |  | 1.167 (1.144, 1.192) |
| NH_4_^+^ | 1.089 (1.065, 1.113) |  | 1.265 (1.230, 1.301) |  | 1.188 (1.158, 1.218) |  | 1.263 (1.228, 1.299) |  | 1.082 (1.059, 1.106) |  | 1.188 (1.159, 1.216) |
| OM | 1.088 (1.071, 1.104) |  | 1.189 (1.168, 1.211) |  | 1.143 (1.124, 1.162) |  | 1.188 (1.167, 1.210) |  | 1.083 (1.067, 1.100) |  | 1.126 (1.108, 1.144) |
| BC | 1.054 (1.045, 1.062) |  | 1.107 (1.096, 1.117) |  | 1.080 (1.070, 1.089) |  | 1.106 (1.096, 1.117) |  | 1.052 (1.043, 1.061) |  | 1.071 (1.061, 1.080) |
| HW11 | 1.019 (1.010, 1.028) |  | 1.013 (1.003, 1.023) |  | 1.019 (1.010, 1.028) |  | 1.012 (1.002, 1.022) |  | 1.018 (1.009, 1.027) |  | 1.014 (1.005, 1.023) |
| CS10 | 1.091 (1.080, 1.102) |  | 1.077 (1.065, 1.089) |  | 1.086 (1.075, 1.098) |  | 1.075 (1.063, 1.088) |  | 1.088 (1.077, 1.099) |  | 1.084 (1.073, 1.095) |

Abbreviations: CMM, cardiometabolic multimorbidity; HR, hazard ratio; CIs, confidence intervals; PM_2.5_, fine particulate matter; NO_3_^-^, nitrate; SO_4_^2-^, sulfate; NH_4_^+^, ammonium; OM, organic matter; BC, black carbon; HW11, heat wave frequency for the definition of daily average temperature equal to or higher than 97.5^th^ percentile for at least 3 consecutive days; CS10, cold spell frequency for the definition of daily average temperature lower than 2.5^th^ percentile with at least 2 consecutive days.

Notes: The main model was fully adjusted for age, gender, body mass index, marital status, education, smoking status, residence type, and region.

^a^ 7368 missing data from CLASS.

**Table S10**. Associations of exposure to extreme temperature events, PM_2.5_ and its constituents with CMM occurrence based on a nested case-control study.

|  | Increment | OR (95%CI) | *p*-value |
| --- | --- | --- | --- |
| PM_2.5_ | 10 μg/m^3^ | 1.110 (1.065, 1.157) | < 0.001 |
| NO_3_^-^ | 1 μg/m^3^ | 1.032 (1.015, 1.049) | < 0.001 |
| SO_4_^2-^ | 1 μg/m^3^ | 1.058 (1.034, 1.082) | < 0.001 |
| NH_4_^+^ | 1 μg/m^3^ | 1.057 (1.030, 1.085) | < 0.001 |
| OM | 1 μg/m^3^ | 1.051 (1.032, 1.071) | < 0.001 |
| BC | 1 μg/m^3^ | 1.031 (1.020, 1.041) | < 0.001 |
| HW11 | 1 day | 1.019 (1.008, 1.030) | 0.001 |
| CS10 | 1 day | 1.035 (1.022, 1.041) | <0.001 |

Abbreviations: PM_2.5_, fine particulate matter; CMM, cardiometabolic multimorbidity; OR, odds ratio; CIs, confidence interval; NO_3_^-^, nitrate; SO_4_^2-^, sulfate; NH_4_^+^, ammonium; OM, organic matter; BC, black carbon; HW11, heat wave frequency for the definition of daily average temperature equal to or higher than 97.5^th^ percentile for at least 3 consecutive days; CS10, cold spell frequency for the definition of daily average temperature lower than 2.5^th^ percentile with at least 2 consecutive days.





**Figure S1**. Flowchart of the current study.

Abbreviations: CMM, cardiometabolic multimorbidity.

Notes: Key information includes one of the cardiovascular and metabolic diseases (heart disease, stroke, diabetes), age, sex, body mass index, marital status, education level, residence type, smoking and drinking status, and hypertension, and residence information.





**Figure S2.** The geographic distribution of monitoring stations of NCEI in the current temperature assessment. Abbreviations: NCEI, National Center for Environmental Information. Notes: the orange triangles denote the stations.


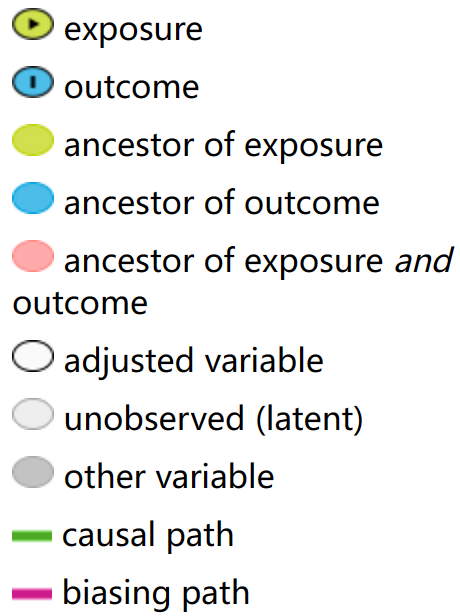

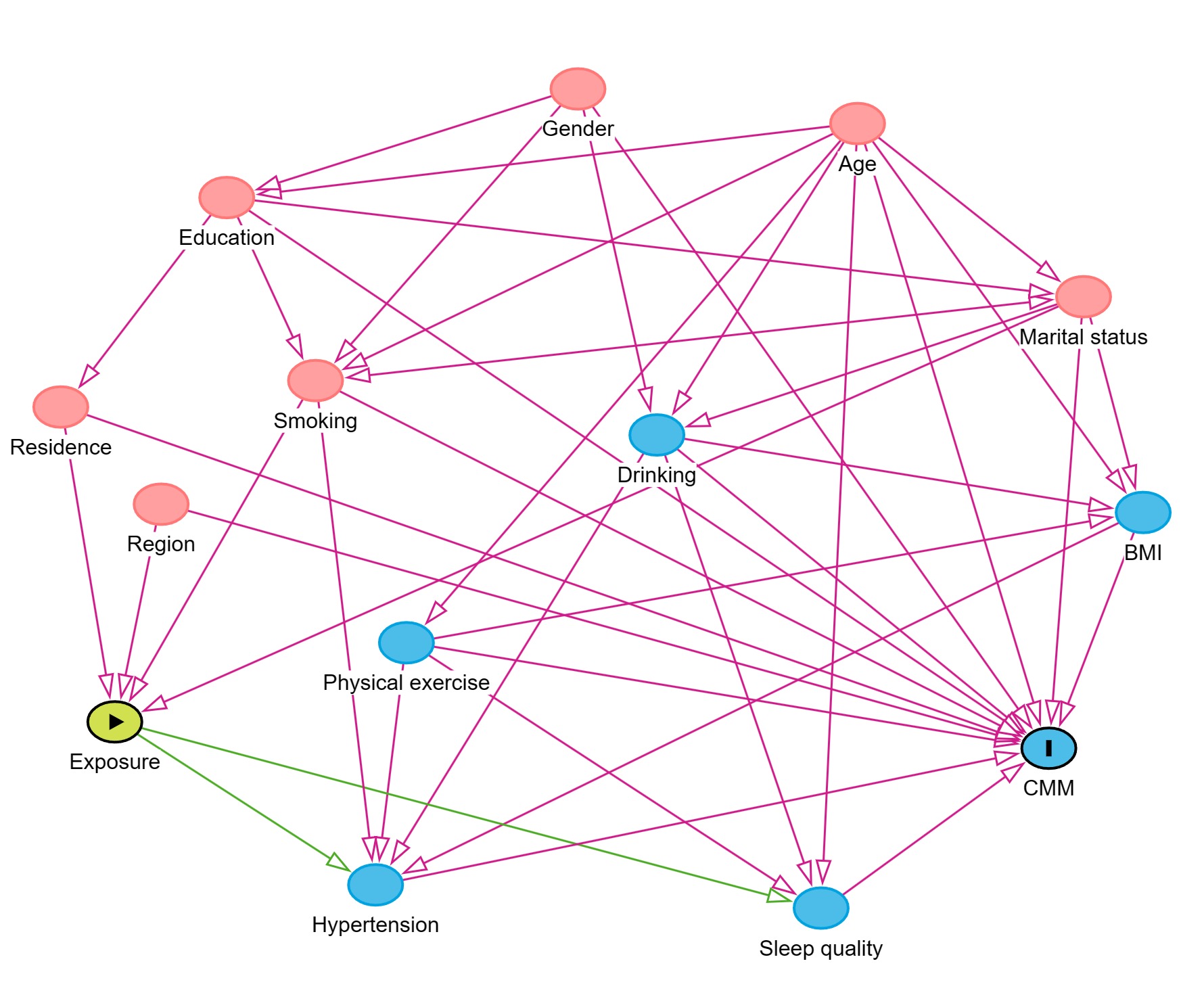


**Figure S3**. Directed Acyclic Graph for the association of exposure to extreme temperature events, PM_2.5_ and its constituents with CMM occurrence.

Abbreviations: CMM, cardiometabolic multimorbidity; BMI, body mass index; PM_2.5_, fine particulate matter.

Notes: Current exposure reflects both extreme temperature events (heatwave or cold spell) and PM_2.5_ with its constituents' pollution.





**Figure S4.** Age distribution of current study participants.


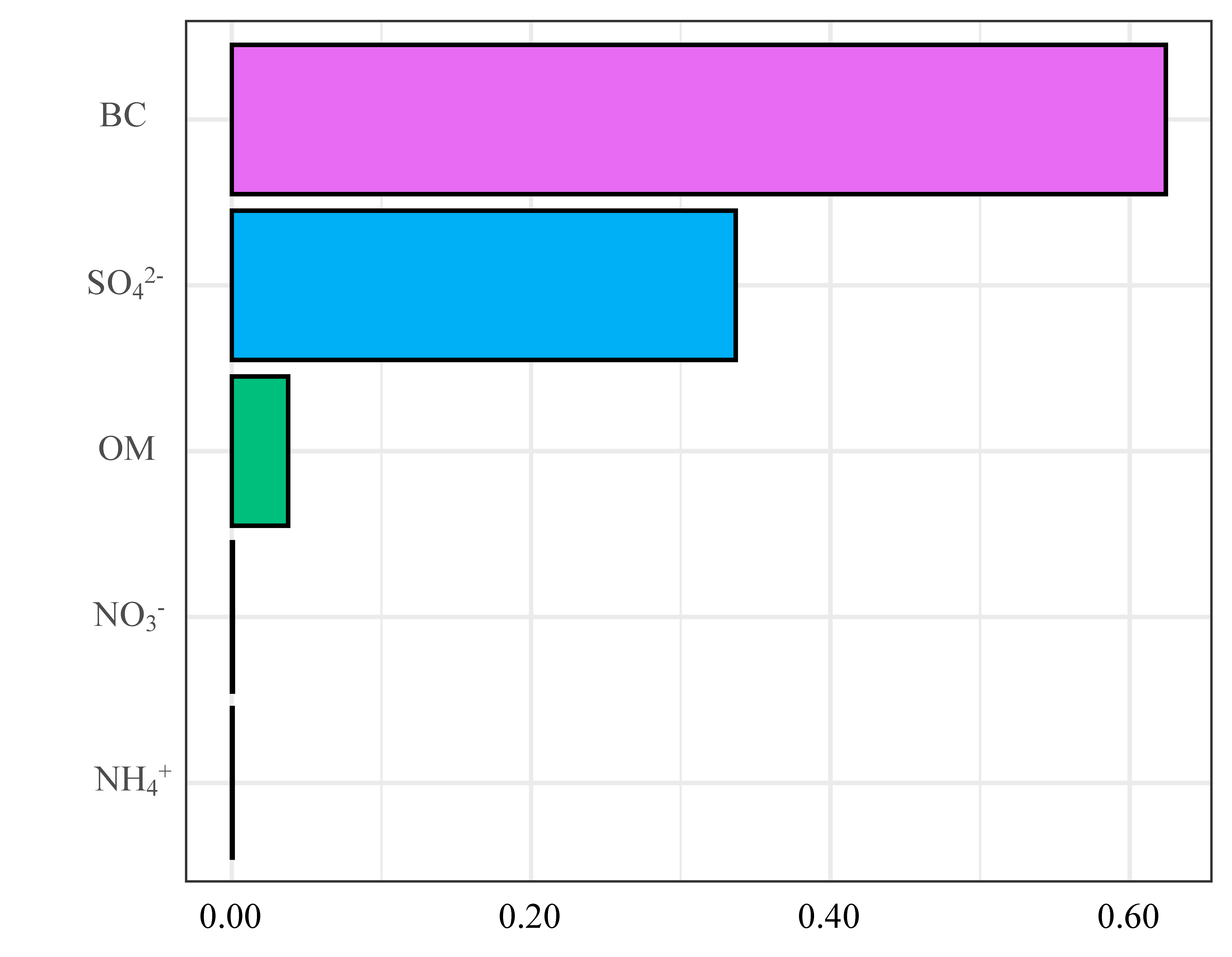


**Figure S5**. The weights of 5-kinds PM_2.5_ constituents mixture based on weighted quantile sum regression in positive direction with CMM occurrence. CMM, cardiometabolic multimorbidity; PM_2.5_, fine particulate matter; NO_3_^-^, nitrate; SO_4_^2-^, sulfate; NH_4_^+^, ammonium; OM, organic matter; BC, black carbon.
